# Temporal Transcriptome Analysis Reveals Dynamic Gene Expression Patterns Driving β-Cell Maturation

**DOI:** 10.3389/fcell.2021.648791

**Published:** 2021-05-04

**Authors:** Tiziana Sanavia, Chen Huang, Elisabetta Manduchi, Yanwen Xu, Prasanna K. Dadi, Leah A. Potter, David A. Jacobson, Barbara Di Camillo, Mark A. Magnuson, Christian J. Stoeckert, Guoqiang Gu

**Affiliations:** ^1^Department of Medical Sciences, University of Torino, Torino, Italy; ^2^Vanderbilt Program in Developmental Biology, Department of Cell and Developmental Biology, Center for Stem Cell Biology, Vanderbilt University School of Medicine, Nashville, TN, United States; ^3^Lester and Sue Smith Breast Center, Baylor College of Medicine, Houston, TX, United States; ^4^Division of Human Genetics, The Children’s Hospital of Philadelphia, Philadelphia, PA, United States; ^5^Institute for Biomedical Informatics, Perelman School of Medicine, University of Pennsylvania, Philadelphia, PA, United States; ^6^Department of Molecular Physiology and Biophysics, Vanderbilt University School of Medicine, Nashville, TN, United States; ^7^Department of Information Engineering, University of Padova, Padova, Italy; ^8^Department of Genetics, Perelman School of Medicine, University of Pennsylvania, Philadelphia, PA, United States

**Keywords:** β-cell maturation, glucose-induced insulin secretion, vesicle release, calcium influx, time-series gene expression, RNA sequencing

## Abstract

Newly differentiated pancreatic β cells lack proper insulin secretion profiles of mature functional β cells. The global gene expression differences between paired immature and mature β cells have been studied, but the dynamics of transcriptional events, correlating with temporal development of glucose-stimulated insulin secretion (GSIS), remain to be fully defined. This aspect is important to identify which genes and pathways are necessary for β-cell development or for maturation, as defective insulin secretion is linked with diseases such as diabetes. In this study, we assayed through RNA sequencing the global gene expression across six β-cell developmental stages in mice, spanning from β-cell progenitor to mature β cells. A computational pipeline then selected genes differentially expressed with respect to progenitors and clustered them into groups with distinct temporal patterns associated with biological functions and pathways. These patterns were finally correlated with experimental GSIS, calcium influx, and insulin granule formation data. Gene expression temporal profiling revealed the timing of important biological processes across β-cell maturation, such as the deregulation of β-cell developmental pathways and the activation of molecular machineries for vesicle biosynthesis and transport, signal transduction of transmembrane receptors, and glucose-induced Ca^2+^ influx, which were established over a week before β-cell maturation completes. In particular, β cells developed robust insulin secretion at high glucose several days after birth, coincident with the establishment of glucose-induced calcium influx. Yet the neonatal β cells displayed high basal insulin secretion, which decreased to the low levels found in mature β cells only a week later. Different genes associated with calcium-mediated processes, whose alterations are linked with insulin resistance and deregulation of glucose homeostasis, showed increased expression across β-cell stages, in accordance with the temporal acquisition of proper GSIS. Our temporal gene expression pattern analysis provided a comprehensive database of the underlying molecular components and biological mechanisms driving β-cell maturation at different temporal stages, which are fundamental for better control of the *in vitro* production of functional β cells from human embryonic stem/induced pluripotent cell for transplantation-based type 1 diabetes therapy.

## Introduction

Pancreatic β cells are functionally defined by their capacity for insulin secretion, stimulated by glucose and other nutrients. Loss of functional pancreatic β cells is the primary cause of diabetes, and researchers have intensively studied β-cell development for the last two decades to generate new therapeutic approaches. Type 1 diabetes results from autoimmune destruction of β cells in the pancreatic islet, whereas the more common type 2 diabetes results from peripheral tissue insulin resistance and β-cell dysfunction. Diabetic patients, particularly those suffering from type 1 diabetes, could potentially be cured through transplantation of new β cells. To this end, several protocols have allowed the production of glucose responsive β-like cells from human embryonic stem/induced pluripotent cells (Kushner et al., 2014; Schiesser and Wells, 2014; Tabar and Studer, 2014). These β-like cells show gene expression, ultrastructural characteristics, and glucose responsiveness, both *in vitro* and *in vivo*, which closely resembling the features of β cells found in pancreatic islets (Pagliuca et al., 2014). However, the production of these cells is limited as the final cell population has about 30–60% β-like cells, and many of the remaining cells are relatively uncharacterized and can be undifferentiated progenitors or other types of unwanted cells (Shahjalal et al., 2018). This low efficiency is partly due to our lack of understanding the signaling pathways that direct β-cell maturation (Kieffer, 2016).

Newly made insulin-expressing cells, in both the human and rodent fetus, are not mature (pre-β or immature β). They secrete two to five times more insulin than adult β cells with basal glucose (<5.6 mM) while lacking robust glucose-stimulated insulin secretion (GSIS) under stimulating (>10 mM) glucose (Adam et al., 1969; Rorsman et al., 1989; Hellerstrom and Swenne, 1991; Bliss and Sharp, 1994; Rozzo et al., 2009). A maturation process converts pre-β cells into mature β cells with low basal insulin secretion but high GSIS. Several molecular mechanisms can promote β-cell maturation: insulin biosynthesis and vesicle packaging are necessary for insulin secretion (Gu et al., 2010; Blum et al., 2012; Goodyer et al., 2012); glucose influx into β cells, glycolysis, and oxidative phosphorylation lead to ATP production, which represses ATP-sensitive potassium channels to induce Ca^2+^ influx and trigger insulin secretion (Hou et al., 2009; Henquin, 2011); intercellular communication controls the coordinated and pulsatile nature of insulin secretion via GAP junctions (Benninger and Piston, 2014) or heterotypic protein interactions (Konstantinova et al., 2007); several nutritional and neural signals are established to control the dose of secretion to properly regulate blood sugar for physiological demands, thus requiring the production and subcellular localization of neural transmitter receptors and effector channels (Osundiji and Evans, 2013). Different transcriptional factors and signaling molecules, including MafA, NeuroD, and calcineurin (Nishimura et al., 2006; Gu et al., 2010; Goodyer et al., 2012), control and promote the maturation processes. Similarly, gene regulatory mechanisms for proper expression of metabolic genes, such as DNA methylases, regulate maturation by controlling proper glucose metabolism (Dhawan et al., 2015). Despite these published studies, many questions on maturation remain unresolved. Specifically, the reported stage of maturation varies from 2 days to 2 weeks after birth in rodents (Nishimura et al., 2006; Rozzo et al., 2009; Blum et al., 2012), and it is unknown which mechanism(s) represent(s) the limiting step for maturation. To this end, several studies compared the gene expression profiles between mature and immature β cells (Nielsen et al., 2004; Jermendy et al., 2011; Blum et al., 2012; Benitez et al., 2014; Hrvatin et al., 2014; Dhawan et al., 2015). Yet, these comparisons did not consider the dynamics from progenitor to mature β cells, which are necessary to distinguish genes associated with β-cell differentiation (production of insulin^+^ cells) and/or maturation (gaining glucose response). Only few studies monitored gene expression of β-cell maturation at multiple stages so far and recently also at single-cell level, using computational methods able to provide a pseudotemporal ordering of the cells (Qiu et al., 2017). However, in these studies, the altered gene expression levels across the stages are analyzed by computational methods for differential expression such as DEseq2 (Love et al., 2014), comparing each time point independently and without considering the temporal profile of each gene. In addition, the biological interpretation of the obtained lists of differentially expressed genes is usually performed through enrichment techniques, which are applied independently from the gene selection step, thus generating potential false positives/negatives. Finally, the expression profiling of the selected genes is typically displayed through heatmaps, mostly dichotomized into immature and mature cells, without characterizing clusters of temporal expression profiles representing the dynamics of β-cell development and maturation through all the stages.

Here, we examined the dynamics of gene expression across six key stages of β-cell maturation, starting from precommitted endocrine progenitors to adult functional β cells. The aim of the study was to determine temporal patterns (TPs) of functional groups of genes that promote newly born, nonfunctional β cells to become functional glucose-responsive β cells. With respect to the previous analyses, a computational pipeline recently published, named FunPat, was applied to build a comprehensive map of the temporal evolution of functional processes and genome-wide candidate markers. Specifically, FunPat combines gene selection, clustering of temporal expression profiles, and functional annotation into a single framework, and it has shown high precision and recall in detecting the correct temporal expression patterns (Sanavia et al., 2015). The resulting dynamic gene expression profiles were then correlated with the temporal development of β-cell properties associated with GSIS, including insulin vesicle biosynthesis, glucose-induced calcium influx, and insulin secretion. Finally, we also provided a comprehensive database describing the biological processes and pathways characterizing β-cell maturation across time. Among them, several established gene products involved in calcium signaling, whose deregulation critically affects homeostasis in insulin-secreting β cells, showed significant temporal correlation between their enhanced expression and maturation, both highlighting potential targets for diseases such as diabetes and providing useful insights for *in vitro* derivation of β cells to develop new therapies.

## Materials and Methods

### Animal Breeding and Use

Mouse usage followed the procedures specified in protocols M/10/168 and M/11/181 approved by the Vanderbilt Institutional Animal Care and Use Committee. *Mip*^*eGFP*^, *Ngn3^*eGFP*^*, and *Rip*^*mCherry*^ mice were from Hara (Hara et al., 2003), Kaestner (Lee et al., 2002), or reported (Zhu et al., 2015), respectively. The stock mice were kept in mostly Bl6/CBA and 129Sv/Ev mixed background with intercrosses (P0). When heterozygous mice were needed, the stock breeders were directly crossed with CD1 mice to produce P1 progeny for pancreatic tissue collection (Charles River). For collecting *Ngn3^*eGFP/eGFP*^* homozygous embryos, intercrosses between P1 mice were utilized. This crossing scheme allows collection of mice with similar genetic background.

### Antibody Staining

Antibody staining followed routine protocols. Rabbit antiamylase (Sigma–Aldrich) and guinea pig anti-insulin (Dako) were used at 1:1,000 dilution. Cy5-conjugated donkey anti–guinea pig and fluorescein isothiocyanate (FITC)–conjugated donkey anti-rabbit (Jackson Immunology) were used at 1:2,000. Hand-picked islets were used for whole-mount staining and imaged with confocal microscopy.

### Cell Sorting, RNA Extraction, Real-Time Polymerase Chain Reaction, and RNA Sequencing

To examine the gene expression dynamics along the maturation steps, we used four β-cell populations: P1 (day 1 after birth) and P4 β cells are immature cells (Blum et al., 2012); P12 and P60 β cells represent newly matured and fully functional β cells, respectively (Nishimura et al., 2006; Blum et al., 2012; Dhawan et al., 2015). Two progenitor pools transcribing *Ngn3* at embryonic stage E15.5 without (*Ngn3^*eGFP/eGFP*^*) or with Ngn3 protein (*Ngn3*^*eGFP*/+^) were included to establish a baseline of gene expression for differentiation. A portion of the progenitors will become β cells (Gu et al., 2002).

EGFP^+^ and mCherry^+^ cells were sorted using Aria III (BD). *Ngn3^*eGFP*^* pancreata were dissociated with trypsin (Gu et al., 2004) and used for the embryonic stages. For postnatal stages, *Mip*^*EGFP*^ and *Rip*^*mCherry*^ islets were first hand-picked, allowed recovering for 2 h in RPMI media [Life Technologies, 10% fetal bovine serum (FBS)], quickly washed with Ca-Mg free Hanks balanced salt solution (HBSS) (Cellgro) once, and dissociated with trypsin. For hand-picking, P1, P4, and P12 pancreata were directly digested with freshly made 1 mg/ml collagenase IV (Sigma–Aldrich) in HBSS. Perfusion was used for P60 pancreata. After digestion (<10 min), pancreatic clusters were quickly washed with RPMI. All solutions/media used throughout islet isolation have non-stimulating glucose at 5.6 mM. RNA was prepared with TRIzol (Life Technologies) and a DNA-free RNA^TM^ kit (Zymo Research). Two hundred monograms total RNAs with RINs greater than 8 (assessed using Agilent Bioanalyzer 2100) were sequenced, with three biological replicates, following Illumina protocols on HiSeq-2000. Real-time polymerase chain reaction (RT-PCR) utilized SYBR-Green (Promega), following manufacturer’s procedures.

### RNA Sequencing Data Preprocessing and Function-Based Pattern Analysis

Raw reads were aligned to the mouse genome (mm10) and transcriptome using STAR version 2.3.0e (Dobin et al., 2013). Data were normalized and quantified with PORT pipeline to determine the relative expression level of each gene^1^. FunPat was used to select differentially expressed genes that share functional annotation and common dynamic expression profiles (Sanavia et al., 2015). Genes were first filtered using the bounded-area method (Di Camillo et al., 2007), which calculates, for each gene, the area of the region bounded by the time series expression profile and a baseline, set at the expression level in E15.5 *Ngn3^*eGFP*/eGFP^* cells as they have no active β cell–specific genes. A *p*-value was assigned to each gene by evaluating the significance of its bounded-area against a null hypothesis distribution described by a log-normal estimated comparing the biological replicates and characterized by mean and standard deviation equal to 1.09 and 0.44, respectively. Applying a Bonferroni correction to the resulting *p*-values, we named two sets of genes, *seeds* and *candidates*, selected considering adjusted and unadjusted *p*-values below 1%, respectively.

Both *seeds* and *candidates* underwent a function-based clustering approach (Di Camillo et al., 2012; Sanavia et al., 2015), which searches for TPs by performing a linear model-based clustering on the expression profiles, after subtracting the baseline, of groups of genes annotated to the same functional term, e.g., a Gene Ontology (GO) term (Ashburner et al., 2000) or a pathway (Kamburov et al., 2011). Each identified TP contains at least a *seed*, and it represents the mean differential expression across time with respect to the baseline, i.e., expression at E15.5 Ngn3*^*eGFP/eGFP*^*. To lower the percentage of false negatives from the bounded-area method while preserving the false discovery rate, the algorithm selects a gene as differentially expressed if it is a *seed* or if it is a *candidate* that belongs to a TP and therefore shares the same biological annotation with at least a *seed* (Sanavia et al., 2015). Intuitively, all the genes associated with the same TP are likely to be differentially expressed as they are highly correlated to the same temporal profile and, as the clustering is functional term-specific, to a common biological function.

### Functional Annotations

In order to achieve a comprehensive coverage of the functional terms and gene annotations currently available, both functional MGI (Mouse Genome Informatics) annotations to 15,939 GO terms from all categories (Biological Process, Molecular Function and Cellular Component)^2^ and 1,628 pathways from Kyoto Encyclopedia of Genes and Genomes (KEGG) and Reactome derived from ConsensusPathDB (Kamburov et al., 2011)^3^ were considered. While ConsensusPathDB already provides pathway annotations with reduced redundancy by mapping physical entities from different source databases to each other, in order to address the high information redundancy affecting GO annotation, FunPat exploits the hierarchical structure of GO database to search the TPs. Specifically, the method first starts from the most specific terms, represented by the leaf nodes in the ontology, and then it removes the differentially expressed genes associated with a significant TP from the annotations of the ancestor nodes, representing more general concepts (Sanavia et al., 2015). Finally, to summarize the results, the GO terms and pathways selected by FunPat were grouped into (1) functional categories related to common ancestor terms and (2) biological processes potentially related to GSIS according to MacDonald et al. (2005) and Benitez et al. (2012) and a manually-defined list of reference GO ancestor terms. Pathways were linked to the GO terms with the most similar meaning and associated with the corresponding common ancestor term. Pathways specifically related to diseases were grouped into a separate functional category named “Disease.”

### Time-Dependent Organization of the Temporal Patterns

To better interpret the main dynamics characterizing the biological mechanisms involved in β-cell maturation, the identified TPs were sorted according to the most representative maturation stage. Specifically, based on the temporal progression *x* = {*x*_1_ = E15.5 Ngn3^eGFP/eGFP^, *x*_2_ = E15.5 Ngn3^eGFP/+^, *x*_3_ = P1, *x*_4_ = P4, *x*_5_ = P12, *x*_6_ = P60} and the TP = {TP(*x*_1_),..,TP(*x*_6_)}, one or more *time break(s)* was(were) assigned to each TP. A specific stage *x*_*i*_ was identified as a *time break* if, observing the pairs {*x*_*i*__–__1_, *x*_*i*_} and {*x*_*i*_, *x*_*i*__+__1_}:

$$\frac{\left|\text{TP}\left(x_{i}\right)-\text{TP}\left(x_{i-1}\right)\right|}{\bar{\text{TP}}}\cup \frac{\left|\text{TP}\left(x_{i+1}\right)-\text{TP}\left(x_{i}\right)\right|}{\bar{\text{TP}}}>0.2$$

i.e., the TP showed in at least one of the pairs an increased or decreased level with a change over 20% with respect to the mean expression of the corresponding pattern. Time breaks were searched starting from E15.5 Ngn3^eGFP/+^, whereas for the last stage (P60) the variation with respect to the time breaks identified in the previous stages was also evaluated. If more than one time break was assigned to the same TP, the break corresponding to the highest expression difference with respect to the baseline was selected, creating at the end five time-dependent groups of TPs. The TPs associated with the same time breaks were then summarized into *main patterns* (MPs) by applying the linear model-based clustering approach used in the FunPat pipeline. In this way, while TPs represent clusters of genes, the MPs are representative of clusters of functional terms, increasing the interpretability of the results.

### Biological Interpretation of the Main Patterns

Each MP was classified into “positive” or “negative” according to the corresponding sign at its most representative time break, e.g., an MP = {MP(*x*_1_),..,MP(*x*_6_)} having the most representative time break at *x*_5_ (i.e., P12) is defined positive if MP(*x*_5_) > 0. Fisher’s exact test was applied to identify the statistically enriched biological functions, i.e., the functional categories or GSIS processes, in two cases: (1) genes belonging only to positive or negative MPs, regardless of timing and (2) focusing on positive or negative MPs and considering the timing of the transcriptional activation or inactivation. For this latter case, in order to have enough genes to compare for each case, the time breaks were grouped in order to represent embryonic (E15.5 Ngn3^*eGFP*/+^), nascent (P1, P4), and older (P12, P60) β cells. The *p*-values resulting from the Fisher’s exact test represent the probability that the observed numbers of genes belonging to a specific case (e.g., “genes belonging to positive MPs” or “genes belonging to a positive MP and mainly activated in P60 β cells”) and annotated with a functional category/GSIS process have resulted from random sampling. Results showing a false discovery rate (FDR)–adjusted *p*-value lower than 5% were considered significant.

### Antibody Staining, Ca^2+^ Imaging, *in vitro* GSIS, Immunoassays, and Transmission Electron Microscopy

Antibody staining followed routine protocols. Rabbit anti-amylase (Sigma–Aldrich) and guinea pig anti-insulin (Dako) were used at 1:1,000 dilution. Cy5-conjugated donkey anti–guinea pig and FITC-conjugated donkey anti-rabbit (Jackson Immunology) were used at 1:2,000 (Zhao et al., 2010). Hand-picked islets were used for whole-mount staining and imaged with confocal microscopy.

After 2-h recovery in RMPI1066 (5.6 mM glucose, 10% FBS), hand-picked islets were used for GSIS, immunoassays, and transmission electron microscopy (TEM). GSIS used basal glucose of 2.8 or 5.6 mM and stimulatory glucose of 20 mM as in Zhao et al. (2010). The percentage of insulin release from starting islets within a 45-min window was assayed. Immature and mature vesicles were classified and quantified according to electron density, with ImageJ. Ca^2+^ imaging followed protocols in Jacobson et al. (2010). All antibodies were from Jackson ImmunoResearch. For all the experimental comparisons, two-sided Student *t*-test was applied when the number of samples was >30; otherwise, Wilcoxon rank-sum test was used. For all the statistical tests, FDR-adjusted *p*-values < 5% were considered significant.

## Results

### Exploring Mip^*eGFP*^ Mice for β-Cell Isolation and Gene Expression Studies

**FIGURE 1:**
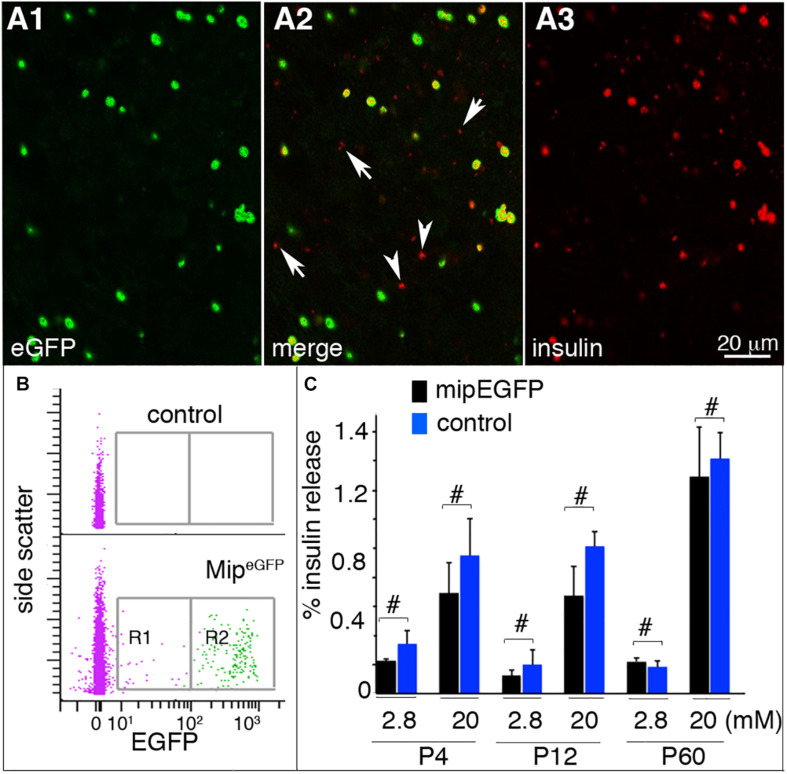
*Mip*^*eGFP*^ islets maintain glucose responsiveness. Hand-picked, dissociated or intact, islets were used for these assays. **(A)** Immunostaining of P4 Mip^*eGFP*^ islet cells. Arrowheads: insulin^+^ cell with undetectable EGFP. Arrows: debris (judged by size) picking up secondary antibodies. **(B)** EGFP analysis in P4 *Mip*^*eGFP*^ islet cells via flow cytometry. R1 and R2 are the EGFP^*Lo*^ and EGFP^*Hi*^ cells collected for RNA-seq. **(C)** GSIS of islets from *Mip*^*eGFP*^ mice and wild-type littermates at P4, P12, and P60. Shown are the percentages of total insulin from starting islets released in 30 min. ^#^*p* ≥ 0.1, Wilcoxon rank-sum test (*n* = 5, number of individual GSIS assays). At least six mice were used for each study, with islets from two to three mice mixed as technical and biological repeats.

Before applying the temporal analysis on both embryonic and postnatal stages, we first checked the utility of the Mip^*eGFP*^ cell–based gene expression for studying β-cell maturation at postnatal stages. Therefore, we examined whether *eGFP* expression labels all the β cells in the *Mip*^*eGFP*^ mice used for β-cell purification with FACS. As reported by Katsuta et al. (2012), the levels of eGFP in insulin^+^ cells greatly varied (Figures 1A,B), appearing as eGFP^High^ and eGFP^Low^ cells. Yet most of the insulin^+^ cells expressed detectable eGFP (1,143/1,216 counted insulin^+^ cells express eGFP; Figure 1A). These data suggested that collecting both eGFP^*High*^ and eGFP^*Low*^ cells (Figure 1B) will provide representative β cells in islets. We also examined whether the *Mip*^*eGFP*^ transgene interferes with endocrine islet function. At P4, P12, and P60, *Mip*^*eGFP*^ islets showed similar GSIS profiles as those of control islets (Figure 1C), suggesting the lack of detectable effects of *Mip*^*eGFP*^ transgene on GSIS.

**FIGURE 2:**
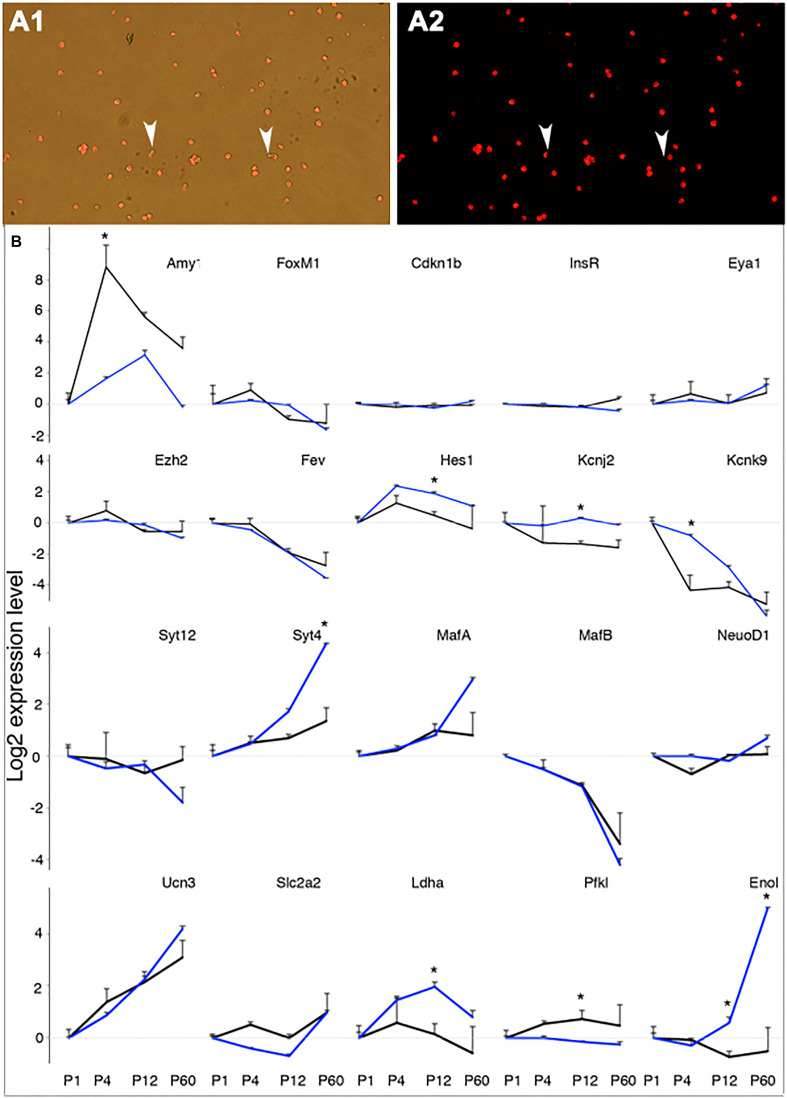
Verification of RNAseq data with RT-PCR in P1-P60 beta cells. The gene expression in P1 beta cells was used as a reference point for other stages. **(A)** An example of sorted P4 beta cells from *Rip*^*mCherry*^ mice. Note the two mCherry-negative cells (arrowheads) in the sorted population. **(B)** The expression levels of 20 genes, assayed with RT-PCR in *Rip*^*mCherry*^+ cells (black lines) and RNAseq in *Mip*^*eGFP*^ cells (blue lines). *p < 0.05, Wilcoxon rank-sum test. For each time point, three RNA preps [each from one (P60) to three mice (P1 to P12)] were used for RT assays.

As further validation of using Mip^*eGFP*^ cell–based gene expression for monitoring β-cell maturation, we examined the expression of 60 genes in Rip^*mCherry*^ β cells with RT-PCR and determined whether the results consistently match the RNA sequencing (RNA-seq) results in *Mip*^*eGFP*^β cells. *Rip^*mCherry*^* mice express mCherry in β cells under the control of a rat *Insulin* 2 promoter and a SV40 polyA. These mice have normal β-cell function and GSIS (Zhu et al., 2015). We purified β cells to ∼98.4% purity from *Rip^*mCherry*^* mice at P1, P4, P12, and P60 (Figure 2A). The set of 60 genes chosen for RT-PCR includes both genes known for β-cell maturation and genes that are not expressed in β cells as positive and negative controls, respectively. We also included genes required for proliferation, differentiation, β-cell electrical activity, vesicular biosynthesis and secretion, stress responses, and metabolism, because of their established roles in β-cell production and GSIS (Figure 2B and Supplementary Figure 1). We randomly picked candidates expressed at both high (such as *Pax6*, *Pdx1*, and *Hsps* that are known in β-cell differentiation and function) and low levels (e.g., *Pax4*, *Ngn3*, and *Ptf1a*, involved in progenitor differentiation and down-regulated in β cells) to better represent the data set. *Hprt* was used as an internal control for PCR.

The expressions of non–β-cell genes, such as *Amy1* (Figure 2B), *Arx*, *Gcg*, and *Ptf1a* (Supplementary Figure 1), displayed disparate patterns between *Mip^*eGFP*^* RNAseq and *Rip^*mCherry*^* RT-PCR results. These findings are consistent with a possibility that exocrine cells cannot be removed at 100% efficiency, and different samples could result in unpredictable acinar contaminations. Indeed, the per-cell levels of *Amy1* in P1 sorted β cells are between 0.5 and 3% of that in total pancreata, consistent with the high purity of the sorted β cells (with ct ∼21 in total pancreas vs. ct ∼27 in sorted β cells). Among the rest of the 55 genes, 33 showed no significant difference in expression at all stages (Figure 2B and Supplementary Figure 1); 17 genes showed similar trends of expression dynamics with one stage that displayed significant differences between the two data sets, including *Hes1*, *Kcnj2*, *Kcnk9*, *Syt4*, *Ldha*, *Pfkl* (Figure 2B), *Dnajb9*, *Fboxo2*, *His2hc31*, *Irx2*, *Kcnj5*, *Kcnk3*, *Rab3a*, *Tmed5*, *Tpt1*, *Vgf*, and *Zcchc12* (Supplementary Figure 1); 5 genes, i.e., *Enol* (Figure 2B) and *Atf3*, *Cbs*, *Hspa1b*, and *Syt14* (Supplementary Figure 1), displayed significant difference at two stages, disrupting the dynamic trends of gene expression (Supplementary Figure 1). Notably, all known genes involved in maturation (*MafA*, *MafB*, *NeuroD*, and *Ucn3*) showed identical expression dynamics between *Mip^*eGFP*^* and *Rip^*mCherry*^* β cells (Figure 2B). These combined findings suggest that most of the gene expression dynamics obtained from *Mip^*eGFP*^* cells reflect that of wild-type β cells.

### Temporal Transcriptome Analysis of β Cells and Progenitors From RNA-Sequencing Data

RNA-seq data were generated for six cell populations from endocrine progenitors (E15.5 Ngn3^eGFP/eGFP^ and E15.5 *Ngn3^eGFP/+^*) to mature β cells monitoring the postnatal stages P1, P4, P12, and P60 (see section “Materials and Methods”). There were 37 million to 85 million raw reads produced per sample. An average of 84.5% of the reads were uniquely mapped. Normalized expression counts were obtained for 39,016 Ensembl genes. Genes with fewer than 10 counts in each stage on average across the biological replicates were filtered out, leaving 18,445 expressed genes. The expression values of these genes were then log2-transformed (zero counts were kept as 0) for further analyses.

The FunPat pipeline identified 4,682 differentially expressed genes (Supplementary Tables 1, 2) across the six cell populations. Forty-five of these genes, including proteases (amylase, trypsin, CPAs, etc.) and nucleases (RNAse 1), were highly expressed in acinar cells (unpublished data). We therefore suspect that these mRNA could come from acinar contamination when FACS can only achieve 98–99% cell purity for the β cells (Supplementary Figure 2A). Indeed, immunoassays showed that young islets could tightly associate with amylase^+^ cells, which make them impossible to remove by hand-picking. Moreover, we could not detect amylase proteins in β cells despite the substantial level of mRNA in sorted β cells (Supplementary Figures 2B,C and Supplementary Table 1). Therefore, these 45 genes were removed for further analysis.

**FIGURE 3:**
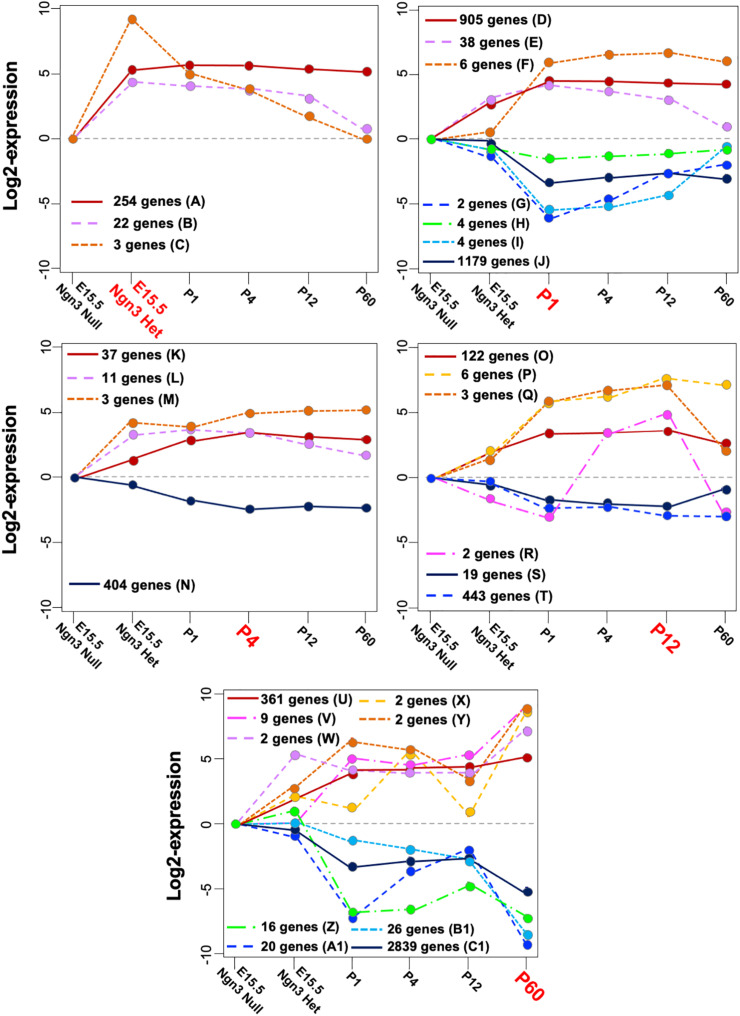
Mature patterns (MPs) identified in maturing β cells. Positive and negative MPs classified according to their time break (highlighted in red) and the dynamic expression across all stages. Note that the line *y* = 0 represents the basal gene expression at E15.5 in *Ngn3*^eGFP/eGFP^ cells that do not express endocrine specific genes. The capital letters are referred to in the MP description in Supplementary Table 1 and in the main text.

Considering the sign of the differential expression over the baseline at their corresponding time breaks, the TPs identified from the remaining genes were summarized into 11 “negative” and 18 “positive” MPs. Overall, more genes related to negative (3,436) rather than to positive (1,201) MPs were identified, as early progenitors are more heterogeneous than maturing β cells. Figure 3 displays the resulting MPs grouped according to the most representative time break. Twenty-one genes clustered alone, and their temporal profiles are reported in Supplementary Figure 3.

Most genes stabilized their transcriptional activity between P1 and P12 (MPs “A,” “D,” “J,” “K,” “N,” “O,” “T,” “U,” and “C1” in Figure 3). Interestingly, 254 genes in MP “A” showed steady-state expression from E15.5 *Ngn3*^*eGFP*/+^ to P60, suggesting that these genes are involved in both β-cell differentiation and function. Several dynamics associated with highest/lowest expression level from the baseline were observed at P60. The positive MPs “U-Y” include known genes regulating cellular stress (*DNAj9* and *Hsps*), membrane permeability (*Lrrc55*), cargo transport (*Ina* and *Neb*), and signaling (*Gdf3*, *Gria 2*, *Prlr*, and *RGS*). On the other hand, the negative MPs “Z-C1” displayed various degrees of decreased expression between P12 and P60, including down-regulation of genes, like *Slc16a1* and *Ldha*, shown to be necessary for β-cell maturation (Ainscow et al., 2000).

More dynamic changes were observed in some MPs with few genes. For example, the 25 genes in MPs “B, C” showed decreased expression after E15.5 *Ngn3*^*eGFP*/+^. Their main positive roles are likely for endocrine differentiation, but not GSIS. Examples include *Ghrl*, *Arx*, and *Irx2* (MP “C”). *Ghrl* suppresses GSIS, whereas *Arx* and *Irx2* are determinants of α cells (Collombat et al., 2003; Petri et al., 2006). Negative MPs “G” and “I” showed decreased expression between *Ngn3*^*eGFP*/+^ and P1, followed by a postnatal increase. These include *Cbs*, *Gabra4*, and *Wdr86* that regulate metabolism and neurotransmission of vesicle fusion (MacDonald et al., 2005; Benitez et al., 2012), consistent with the importance of metabolism for GSIS. MP “P”, showing continuously increased expression between P1 and P60, includes *Ucn3*, shown to stimulate insulin secretion in functional β cells (Blum et al., 2012). MP “R”, showing increased expression between P1 and P12 followed by a decrease at P60, includes *Sycn*, a secretory granule protein acting as Ca^2+^-sensitive regulator of exocytosis (Li et al., 2007), and *Reg1*, involved in proliferation of β cells. Interestingly, *Reg2* is among the 21 single-gene MPs, and it showed its highest expression at P12 as does *Reg1* but with positive differential expression at *Ngn3*^*eGFP*/+^ and P1 (Supplementary Figure 3). The functional implication of *Reg1* in the maturation process is not clear yet.

### Main Functional Terms Characterizing β-Cell Maturation

To better summarize the biological information, the functional terms selected by FunPat were grouped into 17 main functional categories (Table 1) related to common ancestor terms and a manually-defined list of reference GO ancestor terms (Supplementary Data Sheet 2). We then examined through Fisher’s exact test the enrichment of each category in both positive and negative MPs (Table 1) in endocrine progenitors, nascent (P1-P4), and older β cells (P12-P60). Overall, 8 of the 17 functional categories were significantly enriched in both the positive and negative MPs, including “Adhesion, communication, aggregation, and migration,” “binding,” “biological regulation and behavior,” “cellular component organization or biogenesis,” “environmental information processing, response to stimulus, and signaling,” “membrane,” “organelle,” and “unclassified.” These findings showed the co-presence of both positive and negative regulators for β-cell maturation. In the positive MPs, all the eight functional categories (1,558 genes) showed enrichment in P1-P4 β cells, and four of them (364 genes) showed further enrichment in mature P12-P60 β cells. These results suggest that most of the genes positively required for maturation reached their highest expression by P4. Only a small number of genes, 364, need to be expressed in later mature β cells. Interestingly, the negative MPs showed different enrichment profiles; all except the “unclassified” category showed enrichment in mature β cells (Table 1). These results suggest that the down-regulation of the genes preventing β-cell maturation is a slower process. Genes in these groups might represent potential limiting factors for maturation.

**TABLE 1:** The 17 functional categories and their enrichment for either positive or negative main patterns (independent of time breaks).

Functional category	Positive (1,201)	Negative (3,436)
Adhesion, communication, aggregation, migration	8.05E-25* (225)	1 (362)
Binding	1.72E-106* (570)	8.08E-12* (1,145)
Biological regulation and behavior	8.05E-25* (199)	1 (322)
Biosynthesis and catalytical activity	7.37E-05* (322)	0.009* (803)
Cell cycle, proliferation, growth, and death	1 (102)	7.43E-20* (690)
Cellular component organization or biogenesis	1.19E-34* (427)	3.48E-13* (1,000)
Developmental process and reproduction	0.122 (241)	0.054 (690)
Disease	1 (77)	1.38E-12* (464)
Environmental information processing, response to stimulus, and signaling	4.88E-13* (371)	1 (790)
Genetic information process	1 (225)	1.26E-83* (1,719)
Immune system	1 (83)	7.44E-06* (372)
Localization and transport	4.11E-36* (365)	1 (479)
Membrane	2.19E-96* (476)	0.434 (747)
Metabolism	0.129 (356)	2.45E-08* (1,118)
Organelle	2.38E-70* (269)	1.11E-04* (450)
System process	3.20E-42* (168)	1 (130)
Unclassified	4.12E-44* (81)	1 (68)

*Enrichment p-values are listed and marked significant (^∗^) for p < 0.05. The number of genes belonging to each category is reported in parentheses.*

Several functional categories were enriched in either the positive or negative MPs, respectively. “Localization and transport” and “system process” were enriched in the positive MPs. “Cell cycle, proliferation, growth, and death,” “biosynthesis and catalytical activity,” “localization and transport,” and “system process” were specifically enriched in negative MPs, with the most significant enrichments occurring in mature β cells. Identification of the “cell cycle, proliferation, growth, and death” category is consistent with the established studies showing the reduced proliferation in β cells.

### Main GSIS Processes Characterizing β-Cell Maturation

Because of the broad definition of the GO-based functional annotation, the above analyses could not reveal some specific biochemical pathways directing β-cell maturation. We therefore further analyzed the data against manually defined 10 GSIS processes according to known pathways reported in the literature (MacDonald et al., 2005; Benitez et al., 2012). Results were reported in Table 2 and Supplementary Data Sheet 2. For the positive MPs, 6 of the 10 processes displayed significant enrichment in immature β cells, including “calcium-mediated processing,” “GTPase and G-protein activity,” “insulin processing and signaling,” “ion transport and homeostasis,” “membrane potential and ion channels,” and “vesicle-mediated transport and secretion by cell.” Only “calcium-mediated processing,” among other enriched processes, showed further enrichment in mature β cells. These data indicate that most of the genes positively needed for β-cell maturation reached their plateau by P4. Yet, their expression is not sufficient to define maturity, and “calcium-mediated processing” is likely the key molecular mechanism limiting the maturation process among the positive regulators.

**TABLE 2:** Enrichment results on GSIS processes for positive and negative MPs, according to time breaks.

GSIS process	Positive (1,206)			Negative (3,431)		
	E15.5-Het (280)	P1-P4 (958)	P12-P60 (483)	E15.5-Het	P1-P4 (1,396)	P12-P60 (2,961)
Calcium-mediated processing	–	1.52E-18* (56)	0.018* (19)	–	1 (7)	0.825 (63)
Glycolysis, glucose processing, pyruvate metabolism, and TCA cycle	0.908 (9)	1 (30)	1 (6)	–	0.032* (64)	0.186 (82)
GTPase and G-protein activity	0.062 (28)	2.06E-20* (109)	1 (22)	–	1 (39)	0.715 (164)
Insulin processing and signaling	0.908 (9)	1.51E-34* (83)	1 (6)	–	1 (7)	1 (87)
Ion transport and homeostasis	0.908 (16)	1.15E-19* (117)	1 (23)	–	1 (43)	1 (108)
Membrane potential and ion channels	0.908 (10)	4.41E-33* (89)	1 (3)	–	1 (11)	1 (40)
Oxidation-reduction and oxygen-mediated activity	–	1 (34)	1 (13)	–	0.217 (72)	0.323 (162)
Oxoacid metabolic process and Fatty acid activity	1 (6)	1 (18)	1 (8)	–	0.005* (97)	0.718 (161)
Protein tyrosine and serine/threonine kinase activity	1 (3)	0.498 (54)	1 (19)	–	1 (52)	0.003* (193)
Vesicle-mediated transport and secretion by cell	0.908 (15)	1.52E-18* (111)	1 (27)	–	1 (44)	1 (109)

The number of genes belonging to each GSIS process, time break, and main pattern (MP) is reported in brackets. *P < 0.05.

Three GSIS processes resulted enriched in the negative MPs. As expected from previous studies, “glycolysis, glucose processing, pyruvate metabolism, and TCA cycle” and “oxoacid metabolic process and fatty acid activity” belong to this group. Their reduction reached the lowest level by P4 (Table 2). Another process “protein tyrosine and serine/threonine kinase activity” was also enriched in the negative MPs, specifically in mature P12-P60 β cells. This finding is consistent with the notion that hormonal regulation is essential for β-cell function.

### Protein–Protein Interaction Network of Calcium-Mediated Processing

**FIGURE 4:**
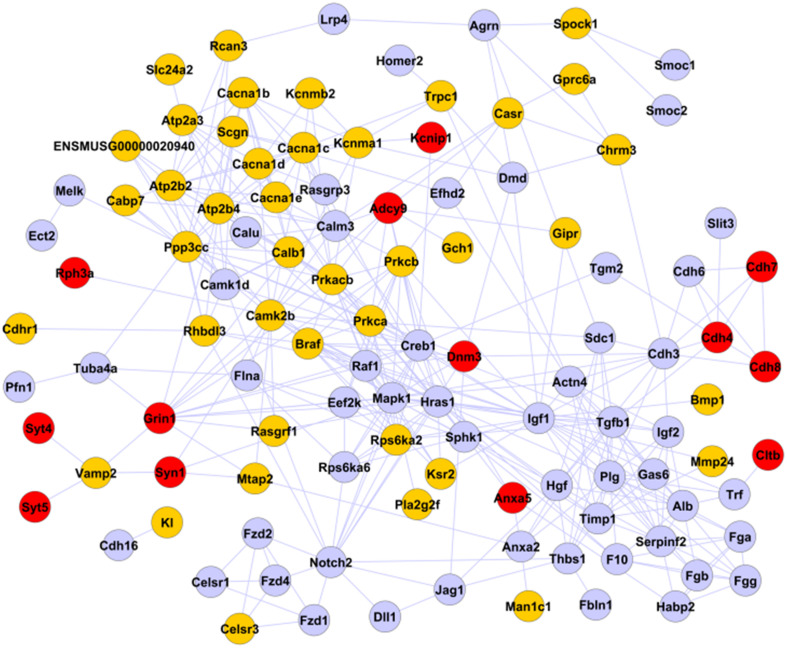
PPI network of calcium-mediated processing. The 109 genes selected and associated with calcium-mediated processing by FunPat correspond to a PPI network of 383 interactions. Gray nodes: genes belonging to negative MPs. Yellow and red nodes: genes belonging to positive MPs. Red nodes: genes belonging to MPs with time breaks at P12 or P60.

We next focused on “calcium-mediated processing,” enriched for positive MPs with time breaks at both nascent and older β cells, form protein–protein interaction (PPI) networks describing potential limiting factors for β-cell maturation. Annotations available from STRING database (von Mering et al., 2003)^4^ were used. 109 of the 142 genes belonging to this process were able to form a PPI network with 383 interactions (Figure 4). 54 of the 109 interacting genes belong to positive MPs, mostly representing channels and G-proteins, consistent with their positive roles in GSIS. The remaining 55 genes assigned to negative MPs were found associated with growth factor signaling (transforming growth factor β and insulin-like growth factor), consistent with lack of significant proliferation of mature β cells. Only 13 genes displayed enriched expression at P12 and P60. These included some vesicular proteins known to be involved in exocytosis process and all linked by *Vamp-2/synaptobrevin*, whose expression level resulted mainly established at birth: *syntaxin-1* (*Syn1*), the NMDA receptor *Grin1*, whose deletion was recently associated with a higher degree of islet GSIS (Marquard et al., 2015), and synaptotagmins *Syt4* and *Syt5* (Gauthier and Wollheim, 2008; Huang et al., 2018). *Syt7* was also found associated with calcium-mediated processing, but it showed no PPIs and highest expression by P1 (Supplementary Table 1). Another complex of *cadherins* (*Cdh4*, *Cdh7*, and *Cdh8*), recently associated with the increase of GSIS activity in β cells (Parnaud et al., 2015), showed their highest transcriptional activity in older β cells.

### Temporal Development of GSIS During β-Cell Maturation

**FIGURE 5:**
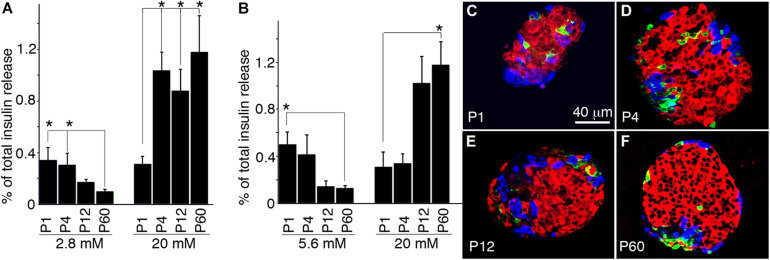
Temporal development of GSIS during β-cell maturation. Hand-picked wild-type islets were used to recover for 2 h before GSIS and immunoassays. For GSIS, the percentages of total insulin from starting islets released in a 45-min window are shown. For all assays, *n* ≥ 4 independent GSIS assays were done. **(A)** Islet GSIS for islets preincubated 2.8 mM glucose for 1 h. **(B)** GSIS after islet preincubated in 5.6 mM glucose for 1 h. **(C–F)** Morphologies of typical isolated islets. Shown are single-confocal planes of whole-mount islets or pancreatic sections that were co-stained for insulin (red), glucagon (blue), and somatostatin (green). *FDR-adjusted *p* ≤ 0.05, Wilcoxon rank-sum test (*n* ≥ 5).

To correlate gene expression dynamics with β-cell maturation, we examined the GSIS of islets at postnatal stages (Figures 5A,B). P1 islets showed higher basal insulin secretion compared to adult islets (2.8 and 5.6 mM glucose), whereas higher glucose did not enhance insulin secretion. P4, P12, and adult islets all significantly responded to high glucose (20 mM) with insulin secretion, but P4 islets resulted not mature, showing higher basal insulin secretion compared to P12/P60 islets. Interestingly, preincubating isolated islets at 5.6 mM glucose before GSIS assays eliminated insulin secretion at high glucose in P4 islets, but not in P12 or P60 islets. The presence of releasable insulin vesicles at low glucose before P12 is consistent with our transcriptional data, showing that most transcripts for insulin vesicle biosynthesis are established before maturation completes (Figure 5 and Supplementary Table 1).

To examine whether GSIS of P1-P60 islets resulted from their intercellular communication with each other, we compared the morphology of hand-picked islets. For islet size, ∼30–100 islets per mice (P1 to P60) were scored. Photographs were taken under a stereoscope, measuring the diameters. At least six mice (three males/three females) were examined at each stage. For looking at the cell-type ratio, ∼20 microscopic fields were scored to examine the percentage of islet cells that express insulin at each stage. At least six mice were scored for each stage. For β cell size, the (insulin^+^ areas)/nucleus (DAPI) was scored with the same fields. The obtained results showed that the size and percentage of β cells increase as they age (Figures 5C–F). Yet they all displayed similar morphology, with β cells clustered in the central region and other cell types in the periphery. Consistently, the gene annotations linked to the functional category “Cell adhesion, communication, aggregation, and migration” are enriched for positive MPs reaching steady state within P4 (Table 2). Therefore, islet organization alone may not account for the different GSIS properties of islets at different ages. Neither does the islet cell-type composition, because P4 and P12 islets display similar β cell/endocrine ratios yet have different maturity.

### Vesicle Biosynthesis in β Cells Correlates With the Temporal Expression of Vesicular Genes

**FIGURE 6:**
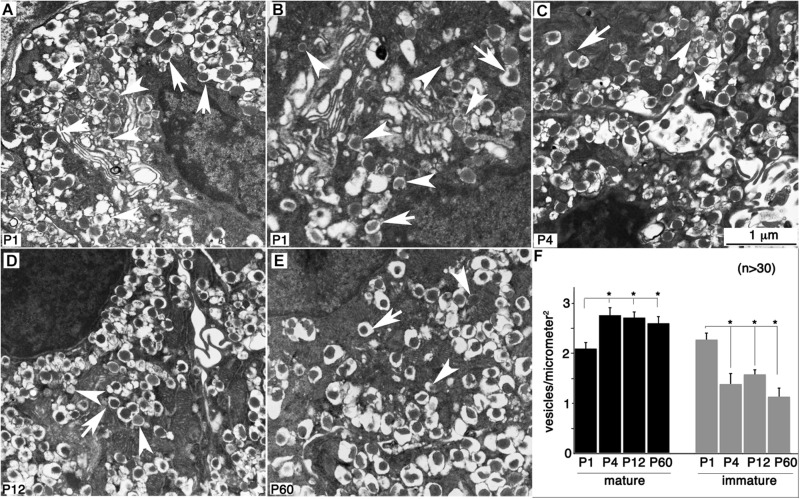
Normal-looking vesicles are present in immature β cells. Hand-picked islets were fixed and examined with TEM. **(A,B)** Two P1 β cells showing different status of vesicular packaging. **(C–E)** Representative images of P4, P12, and P60 β cells. Arrows point to normal-looking “mature” vesicles. Arrowheads point to “immature” vesicles with less dense cores. **(F)** The levels of mature and immature vesicles in β cells are established by P4 based on quantitation with at least 26 microscopic views (1–3 β cells in each view) counted. Images used were from different blocks of at least four mice at each stage. *p < 0.05, two-sided Student *t*-test (*n* > 30).

Previous findings suggested that proper vesicular packaging contributes to β-cell maturation (Blum et al., 2012; Goodyer et al., 2012). As our data showed that transcripts for most vesicular components reached a plateau in their expression by P4 (Table 2 and Supplementary Table 1), we determined the vesicular density in β cells during their maturation process. Indeed, a few P1 β cells showed well-defined mature vesicles with dense-core insulin crystals (Figure 6A), whereas others had mostly immature vesicles with electron-light core (Figure 6B) consistent with the results previously reported in the literature (Blum et al., 2012). By P4, most β cells had mature vesicles of similar appearance (Figures 6C–E), in terms of both vesicle density and morphology (Figure 6F). These results, combined with the temporal sequence of GSIS development, suggest that producing morphologically normal vesicles precedes maturation by over a week. These findings are also consistent with the fact that post-transcriptional regulation does not prominently regulate the production of vesicular proteins, because the appearance of the vesicles structure temporally coincides with gene transcription up-regulation.

### Glucose-Induced Ca^2+^ Influx Is Established in Immature β Cells

The PPI network of the calcium-mediated process also included several genes encoding channel proteins and metabolic enzymes reaching a plateau of expression before P4 (Figure 3 and Table 2). We therefore examined glucose-induced Ca^2+^ influx in islets at different stages. Because Ca^2+^ influx depends on proper glucose transport, metabolism, oxidative phosphorylation, and activation/inactivation of multiple channels, proper Ca^2+^ activity in β cells will likely reveal the production and assembly of all protein complexes and pathways involved in these processes.

**FIGURE 7:**
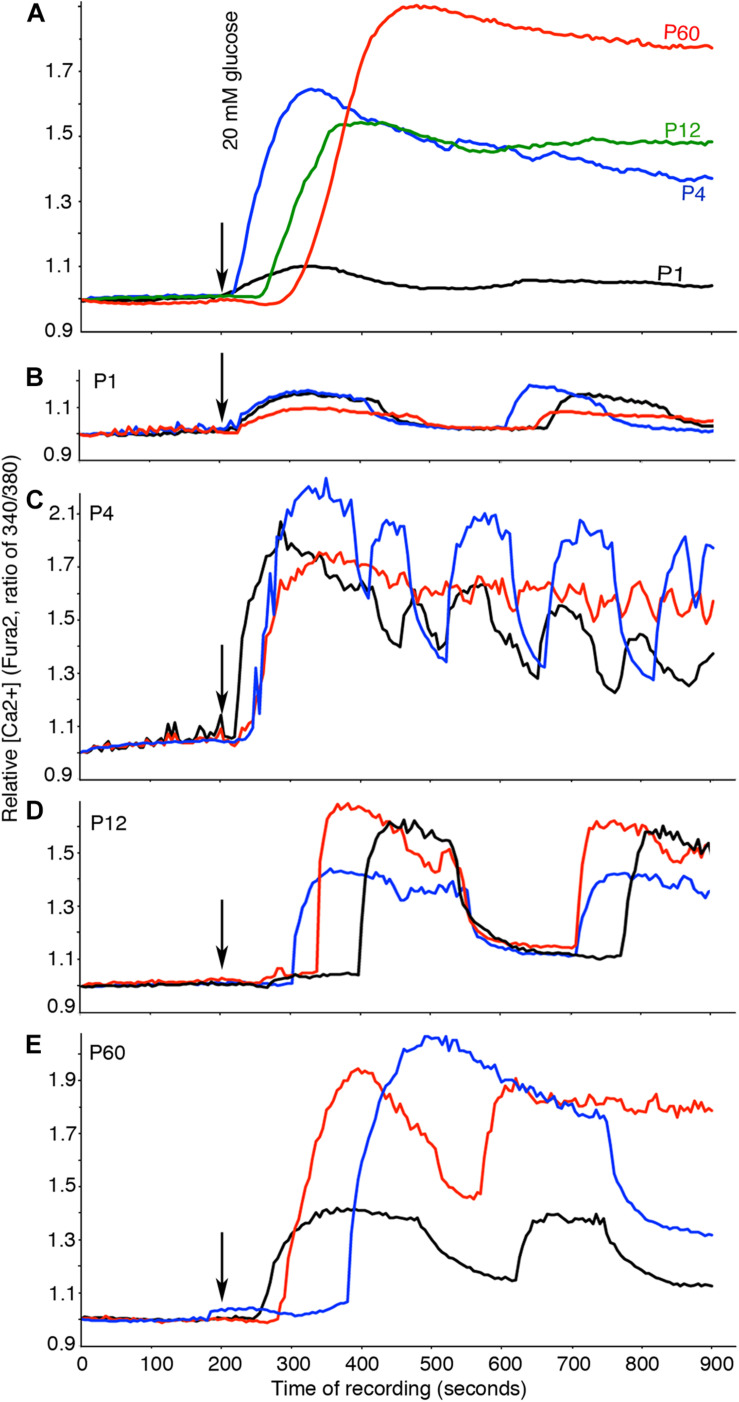
Glucose-induced Ca^2+^ influx is established in immature β cells. Free Ca^2+^ was recorded by Fura2 fluorescence. Basal glucose was used for the first 200 s, and then 20 mM glucose was used to stimulate islets. **(A)** Average Ca^2+^ responses of islets at each stage (*n* ≥ 69). **(B–E)** Examples of oscillating Ca^2+^ in three different islets. The color of lines here indicate different islets of same stages. The numbers of mice used were as follows: P1: 10; P4: 10; P12: 10; P60: 4.

P1 islets displayed very low glucose-induced Ca^2+^ influx (Figure 7A), although they showed recognizable oscillating patterns, a property of β cells (Figure 7B). P4 to P60 islets showed significantly higher glucose-induced Ca^2+^ influx than P1 islets, with β-cell specific oscillations (Figures 7C–E). These findings suggest that the molecular machineries for glucose transport, metabolism, ATP production, ATP-mediated blockage of K^+^ channels, and voltage-gated Ca^2+^ channels are already present in immature β cells at P4. As the mRNAs coding for the proteins involved in the above processes also reached a plateau at P4, these findings indicate that post-transcriptional regulation is not prominently involved in controlling the production of these proteins. Finally, a notable and recurrent difference between immature and mature islets was observed in the 1- to 2-min delay between the applications of high glucose to the Ca^2+^ influx in mature islets (*P* < 0.001, Figure 7A). The reason and significance of this delay are not clear yet.

## Discussion

Our multi-staged transcriptome analysis using FunPat pipeline revealed several candidate genes, dynamic trends, and biological processes able to distinguish genes required for β-cell differentiation (generating insulin^+^ cells) and/or maturation (insulin^+^ cells gaining proper glucose response). Compared with the several pairwise gene expression analyses (Jermendy et al., 2011; Blum et al., 2012; Dhawan et al., 2015), our studies confirmed the importance of proper metabolism, calcium signaling, and vesicular biosynthesis for β-cell maturation. The multi-stage dynamic analysis allowed us to reveal several previously unrecognizable features but also to confirm the expression patterns of several well-recognized markers for predicting β-cell maturation (Benitez et al., 2012) including *MafA* and *Ucn3*, found also in other recent studies at a single-cell level (Qiu et al., 2017; Augsornworawat et al., 2020).

Besides the nature of the transgene, we have considered several variables that could affect our gene expression, including the islet isolation, dissociation (Khan et al., 2016), and mechanical sorting (Beliakova-Bethell et al., 2014). All these processes can in theory alter the gene expression. Yet, our RNAseq-based results directly correlate with tissue-staining–based gene expression, such as *MafA* (Hang et al., 2014), *NeuroD1* (Gu et al., 2010), and *Ucn3* (Blum et al., 2012), suggesting that these technical issues will unlikely invalidate most of the gene expression patterns. It is worth considering that these findings are based on mouse models and that there are differences with human β cells. For example, *MafB* is expressed in human but not mouse β cells (Arda et al., 2016; Xin et al., 2016; Tritschler et al., 2017), whereas *Ucn3* expression is much higher in mouse than human β cells (Xin et al., 2016). However, these observations have been made analyzing β cells by considering no more than two maturation stages; therefore, we believe that our study will be useful for future comparisons with temporal gene expression data from human β cells.

Our RNAseq-based studies could not address whether post-transcriptional regulation prominently regulates β-cell maturation. However, by examining the presence of the vesicular structures and biochemical pathways for insulin secretion and Ca^2+^ influx, we found that the appearance of required proteins closely matched the dynamic activation of gene transcription. These findings suggest that translational regulation is not a major regulatory mechanism for most of the genes involved in β-cell maturation and GSIS.

One of our main conclusions is that the expression of most maturation genes reaches a plateau before P4. Many of these gene products are involved in insulin biosynthesis, signal transduction of transmembrane receptors, vesicle transport, and calcium-mediated processing (Table 2, positive MPs). Indeed, producing Ca^2+^-related proteins or signal transducers is likely needed for proper stimulus-secretion coupling. Such transcription profiles correspond to detection of significantly higher density of mature vesicles (Figure 6F) and glucose-induced Ca^2+^ influx (Figure 7A) in P4 with respect to P1.

In addition, incubating P4 β cells with low basal glucose (2.8 mM, Figure 5A) led to significant insulin secretion when glucose stimulus was switched to 20 mM, but not when basal glucose levels were increased (5.6 mM, Figure 5B). This result suggests that repressing insulin secretion at low glucose could be a limiting step for β-cell maturation. Indeed, incubating immature β cells with higher basal glucose appears to deplete the releasable vesicle pool so that switching to higher glucose could no longer trigger further insulin secretion, as already reported (Blum et al., 2012). The implications of these observations are as follows: (1) vesicles in immature β cells, even if morphologically normal-looking (Figure 6), are not equally releasable, a feature that has been proven by real-time secretion assays (Hou et al., 2012; Hoboth et al., 2015); (2) insulin vesicles need to desensitize themselves from glucose-derived signals to abstain from releasing at basal glucose in the maturation process. This could theoretically be achieved by limiting the Ca^2+^ influx at basal glucose or modulate their Ca^2+^ sensitivity, or both. In support of this idea, enrichment analysis found only “calcium-mediated processing” to be significant among the GSIS processes for positive MPs characterizing both immature and mature β cells (Table 2). The corresponding MPs and PPI network (Figures 3, 4) highlight the interaction of synaptotagmins and cadherins at later stages of maturation, suggesting a possible role in controlling vesicle release and Ca^2+^ influx (Huang et al., 2018).

Finally, the transcriptome data analysis also highlighted the importance of negative GSIS regulation in β-cell maturation, generally represented by MPs showing an expression decrease between P12 and P60. Focusing on GSIS processes, enrichment analysis found only “protein tyrosine and serine/threonine kinases activity” to be significant for negative MPs at late maturation stages (Table 2), suggesting unappreciated phosphorylation processes linked with vesicle biosynthesis and release. This study can be considered a resource for further integrations/comparisons to human β-cell development from embryonic or induced-pluripotent stem cells (Weng et al., 2020). The complete database of TPs, genes, and functional terms available in Supplementary Tables 1, 2 will aid future studies to better characterize the regulatory role of these genes and critical steps for *in vitro–*derived functional β cells.

## Data Availability Statement

The dataset presented in this study can be found at ArrayExpress repository (https://www.ebi.ac.uk/arrayexpress/) with the accession number E-MTAB-2266.

## Ethics Statement

The animal study was reviewed and approved by the Vanderbilt University Animal Care and Use Committee.

## Author Contributions

TS, GG, CS, and MM conceived the work and designed the experiments. TS, GG, and CS wrote the manuscripts, with help from all listed authors. TS, EM, BD, and CS performed all the bioinformatics analysis. CH, YX, and LP isolated the cells and performed the RNA preparations and islet characterization. PD and DJ performed the Ca^2+^ imaging. All authors contributed to the article and approved the submitted version.

## Conflict of Interest

The authors declare that the research was conducted in the absence of any commercial or financial relationships that could be construed as a potential conflict of interest.

## Footnotes

1 https://github.com/itmat/Normalization

2 http://www.informatics.jax.org/function.shtml

3 http://cpdb.molgen.mpg.de/MCPDB

4 http://string-db.org/
