# Supplementary material for: Temporal Transcriptome Analysis Reveals Dynamic Gene Expression Patterns Driving β-Cell Maturation
Source: Front Cell Dev Biol. 2021 May 4;9:648791. doi: 10.3389/fcell.2021.648791 (PMC8129579; doi:10.3389/fcell.2021.648791)
Supplement: Supplementary file 1 [file Data_Sheet_1.PDF]

Supplementary Material

Supplementary Figure 1. Verification of RNAseq data with Real-time RT-PCR in P1-P60 beta cells. The gene expression in P1 beta cells was used as a reference point for other stages. The expression levels of 40 genes, assayed with RT-PCR in Rip$^{mCherry+}$ cells (black lines) and RNAseq in Mip^{eGFP} cells (blue lines). For each time point, three RNA preps [each from one (P60) to three mice (P1 to P12)] were used for RT assays.

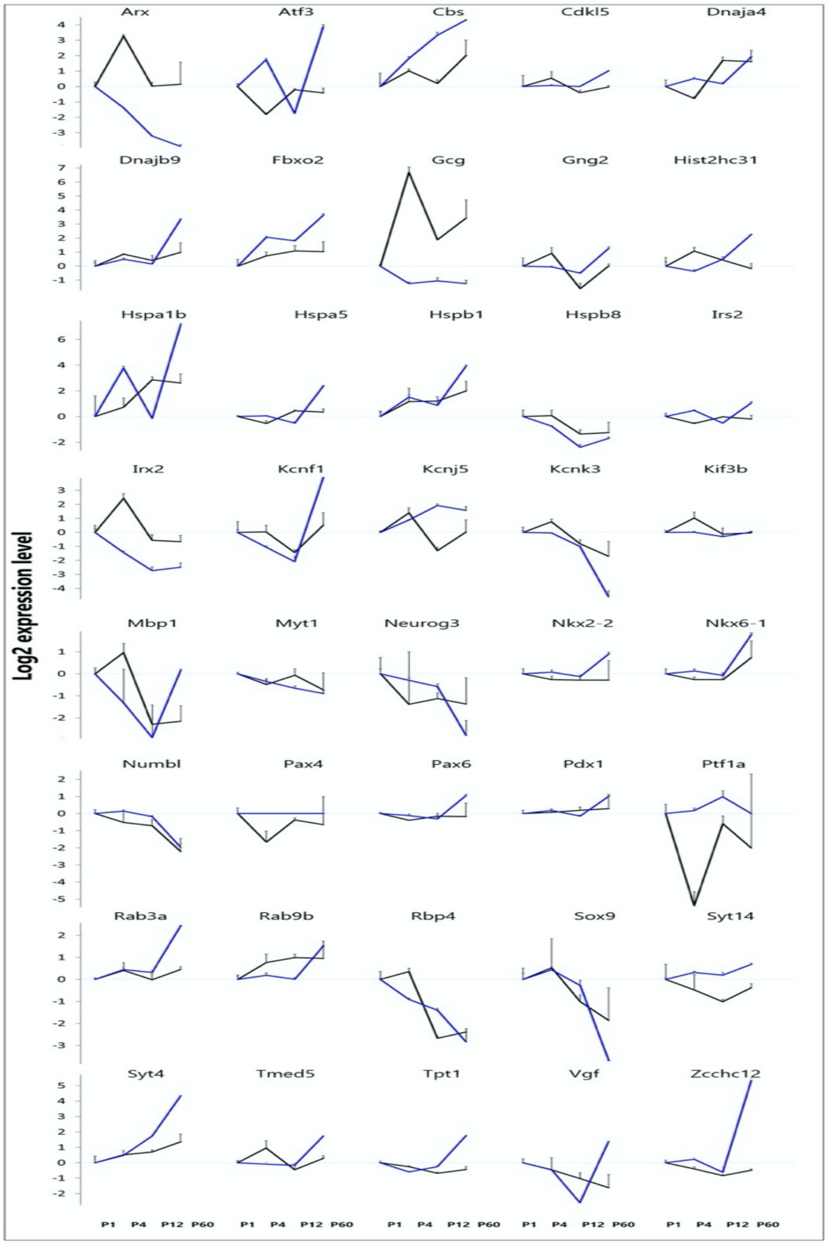

Supplementary Figure 2. Potential contamination of sorted beta cells with exocrine tissue. (A) Visualization of purified eGFP+ cells. Note the presence of some eGFP^{low} cells (red arrows) and rare eGFP- cells (white arrow). Co-staining of amylase and insulin in hand-picked islets. (B) Note that at P1, several amylase+ cells were surrounded by insulin+ beta cells, making them impossible to separate with hand-picking. (C) At P12, only occasionally can single amylase+ cells be detected in some islets.

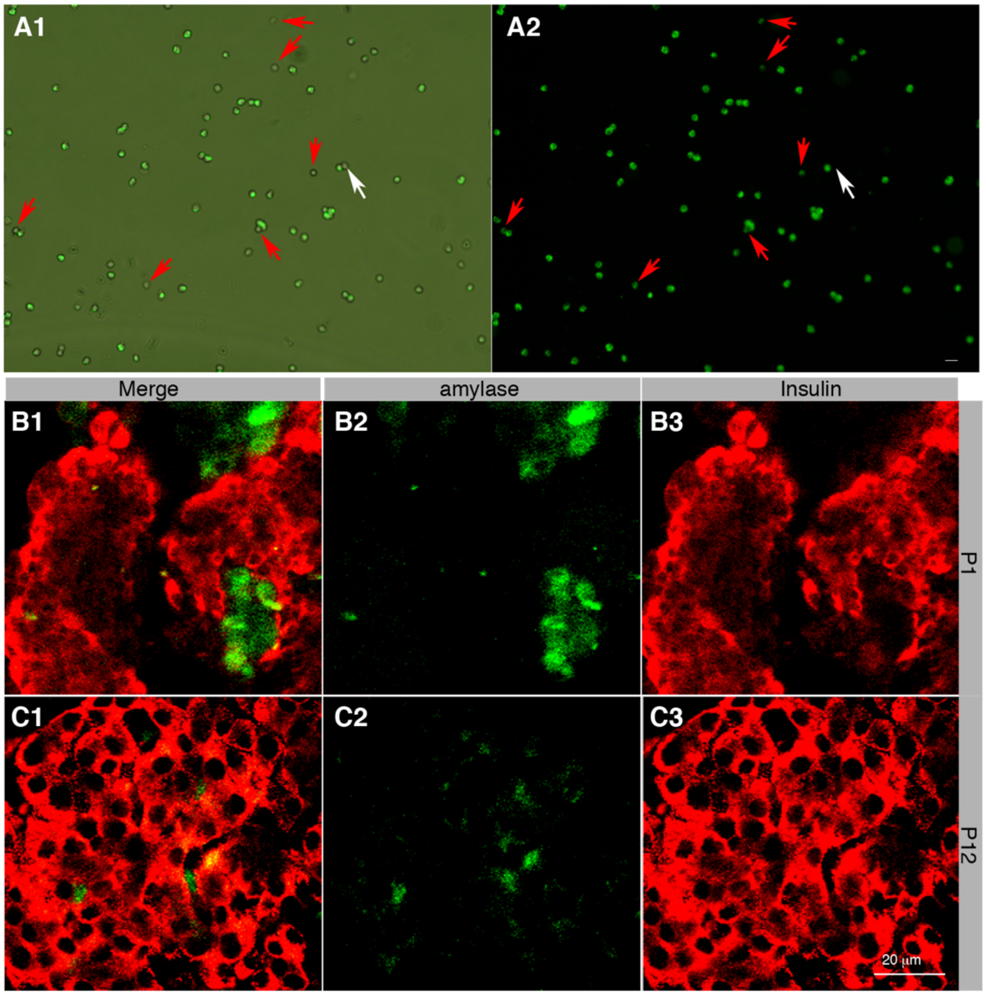

Supplementary Figure 3. Singleton gene profiles from the analysis pipeline. Plot of the temporal profiles of the 21 genes remaining as singletons after the clustering to identify the main patterns. The singletons are classified according to their time-break (highlighted in red).

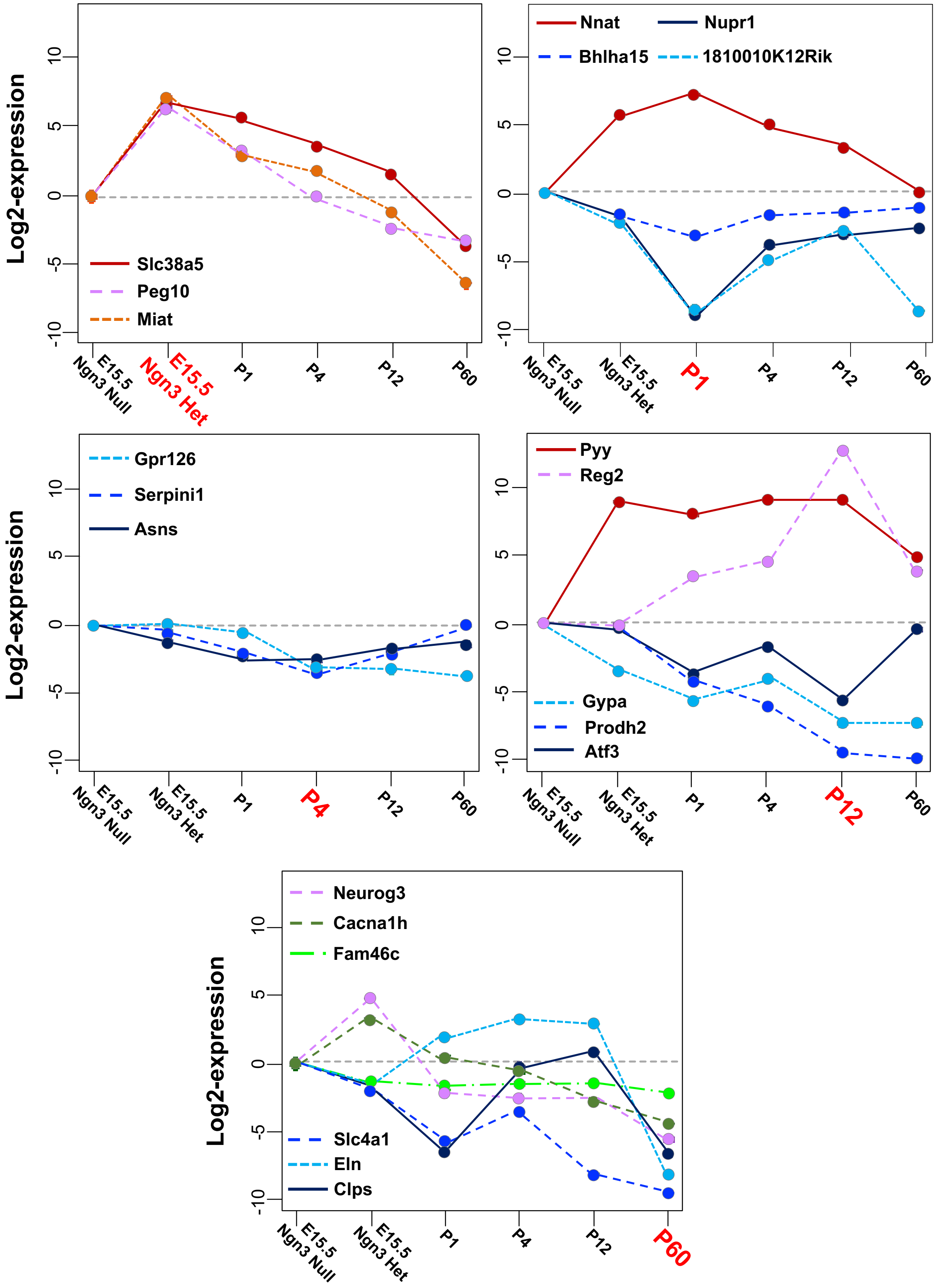

Legend Supplementary Tables

Supplementary Table 1 Differentially expressed genes selected by FunPat pipeline were listed. Column A: gene symbols; columns B-G: log2-transformed expression levels in E15.5 *Ngn3*$^{eGFP/eGFP}$, E15.5 *Ngn3*$^{eGFP/+}$ heterozygous, P1, P4, P12, and P60 beta cells. Column H: p value of differential gene expression resulting from the bounded-area method. Columns I-M: MPs with related time break, sign (e.g. positive or negative) and associated GSIS processes and Functional categories defined according to common ancestor terms. For the classification, pathways were linked to the GO terms with the most similar meaning and associated to the corresponding common ancestor term. See also the Appendix supplementary file for further details on the annotations to Functional categories and GSIS processes. Since KEGG also provides pathways specifically related to diseases and not linkable with any specific GO term, these were grouped into a separate functional category named "Disease" (see http://www.genome.jp/kegg-bin/show_organism? menu_type=pathway_maps&org=mmu). Note that some genes have multiple MPs and time breaks, indicating that they likely show more than one main dynamic expression change across the temporal profile. Moreover, multiple MPs and GSIS/Functional categories can be assigned to each gene since the genes can belong to multiple biological processes. Abbreviations: ACAM=Adhesion, communication, aggregation and migration, Bin=Binding; BRB=Biological regulation and behavior; BCA=Biosynthesis and Catalytical activity; CPGD=Cell cycle, proliferation, growth and death; CCOB =Cellular component organization or biogenesis; DPR=Developmental process and reproduction; Di=Disease; EPRSS=Environmental information processing, response to stimulus and signaling; GIP=Genetic Information process; IIS=mmune system; LT=Localization and transport; Mem=Membrane; Met=Metabolism; Or=Organelle; SP=System process; CMP=Calcium-mediated processing; GGPMTC=Glycolysis, Glucose processing, Pyruvate metabolism and TCA cycle; GGA=GTPase and G-protein activity; IPS=Insulin processing and signaling; ITH=Ion transport and homeostasis; MPIC=Membrane potential and Ion channels; OOMA=Oxidation-reduction and Oxygen-mediated activity; OMPFAA=Oxoacid metabolic process and Fatty acid activity; PTSKA=Protein tyrosine and serine/threonine kinase activity; VTSC=Vesicle-mediated transport and secretion by cell.

Supplementary Table 2 Extended version of Supplementary Table 1. Columns A-C: Ensembl IDs, chromosome locations, and gene symbols. Columns D-H: log2-transformed differential expression levels in E15.5 *Ngn3*$^{eGFP/+}$ heterozygous, P1, P4, P12, and P60 beta cells compared to E15.5 *Ngn3*$^{eGFP/eGFP}$ null cells. Column I: p value of differential gene expression resulting from the bounded-area method. Columns J-P: MPs with related time break, sign (e.g. positive or negative) and associated GSIS processes, Functional categories defined according to common ancestor terms, Gene Sets (i.e. GO terms and pathways) IDs and definitions. See also the Appendix supplementary file for further details on the annotations to Functional categories and GSIS processes. Since KEGG also provides pathways specifically related to diseases and not linkable with any specific GO term, these were grouped into a separate functional category named "Disease" (see http://www.genome.jp/kegg-bin/show_organism? menu_type=pathway_maps&org=mmu). Note that some genes have multiple MPs and time breaks, indicating that they likely show more than one main dynamic expression change across the temporal profile. Moreover, multiple MPs and GSIS/Functional categories can be assigned to each gene since the genes can belong to multiple biological processes. Abbreviations: ACAM=Adhesion, communication, aggregation and migration, Bin=Binding; BRB=Biological regulation and behavior; BCA=Biosynthesis and Catalytical activity; CPGD=Cell cycle, proliferation, growth and

death; CCOB =Cellular component organization or biogenesis; DPR=Developmental process and reproduction; Di=Disease; EPRSS=Environmental information processing, response to stimulus and signaling; GIP=Genetic Information process; IIS=mmune system; LT=Localization and transport; Mem=Membrane; Met=Metabolism; Or=Organelle; SP=System process; CMP=Calcium-mediated processing; GGPMTC=Glycolysis, Glucose processing, Pyruvate metabolism and TCA cycle; GGA=GTPase and G-protein activity; IPS=Insulin processing and signaling; ITH=Ion transport and homeostasis; MPIC=Membrane potential and Ion channels; OOMA=Oxidation-reduction and Oxygen-mediated activity; OMPFAA=Oxoacid metabolic process and Fatty acid activity; PTSKA=Protein tyrosine and serine/threonine kinase activity; VTSC=Vesicle-mediated transport and secretion by cell.
