# Supplementary material for: Temporal Transcriptome Analysis Reveals Dynamic Gene Expression Patterns Driving β-Cell Maturation
Source: Front Cell Dev Biol. 2021 May 4;9:648791. doi: 10.3389/fcell.2021.648791 (PMC8129579; doi:10.3389/fcell.2021.648791)
Supplement: Supplementary file 2 [file Data_Sheet_2.PDF]

Functional categories

Associations between the defined functional categories and the ancestor terms in GO. BP = Biological Process, MF = Molecular Function, CC= Cellular Components. For each category, the numbers of GO terms/pathways and associated genes are reported in brackets.

Adhesion, communication, aggregation and migration (129 terms, 587 genes)

GOID	Ontology	Term
GO:0022610	BP	biological adhesion
GO:0098636	CC	protein complex involved in cell adhesion
GO:0030054	CC	cell junction
GO:0050839	MF	cell adhesion molecule binding
GO:0098631	MF	protein binding involved in cell adhesion
GO:0030370	MF	intercellular adhesion molecule-3 receptor binding
GO:0071135	CC	alpha7-beta1 integrin-focal adhesion kinase complex
GO:0071065	CC	alpha9-beta1 integrin-vascular cell adhesion molecule-1 complex
GO:0098743	BP	cell aggregation
GO:0030155	BP	regulation of cell adhesion
GO:0034330	BP	cell junction organization
GO:1901888	BP	regulation of cell junction assembly
GO:1903391	BP	regulation of adherens junction organization
GO:0007154	BP	cell communication
GO:0010646	BP	regulation of cell communication
GO:0040011	BP	locomotion
GO:0040012	BP	regulation of locomotion
GO:0008037	BP	cell recognition
GO:0045202	CC	synapse
GO:0006928	BP	movement of cell or subcellular component
GO:0033622	BP	integrin activation
GO:0033623	BP	regulation of integrin activation
GO:0045499	MF	chemorepellent activity
GO:0031252	CC	cell leading edge

Binding (157 terms, 1715 genes)

GOID	Ontology	Term
GO:0005488	MF	binding
GO:0051098	BP	regulation of binding

Biological regulation and behavior (92 terms, 521 genes)

GOID	Ontology	Term
GO:0065007	BP	biological regulation
GO:0007610	BP	behavior
GO:0001775	BP	cell activation
GO:0048511	BP	rhythmic process
GO:0098772	MF	molecular function regulator

Biosynthesis and Catalytical activity (275 terms, 1125 genes)

GOID	Ontology	Term
GO:0009058	BP	biosynthetic process
GO:0003824	MF	catalytic activity
GO:0009889	BP	regulation of biosynthetic process
GO:0050790	BP	regulation of catalytic activity
GO:0030234	MF	enzyme regulator activity
GO:0005085	MF	guanyl-nucleotide exchange factor activity
GO:1902494	CC	catalytic complex

Cell cycle, proliferation, growth and death (306 terms, 792 genes)

GOID	Ontology	Term
GO:0097189	CC	apoptotic body
GO:0043293	CC	apoptosome
GO:0007049	BP	cell cycle
GO:0008219	BP	cell death
GO:0008283	BP	cell proliferation
GO:0040007	BP	growth
GO:0022403	BP	cell cycle phase
GO:0010941	BP	regulation of cell death
GO:0051726	BP	regulation of cell cycle
GO:0042127	BP	regulation of cell proliferation
GO:0040008	BP	regulation of growth
GO:0030496	CC	midbody
GO:0005819	CC	spindle

Cellular component organization or biogenesis (175 terms, 1427 genes)

GOID	Ontology	Term
GO:0071840	BP	cellular component organization or biogenesis
GO:0032991	CC	macromolecular complex
GO:0051128	BP	regulation of cellular component organization
GO:0044087	BP	regulation of cellular component biogenesis
GO:0007163	BP	establishment or maintenance of cell polarity
GO:0051301	BP	cell division
GO:0051302	BP	regulation of cell division
GO:0045177	CC	apical part of cell
GO:0032878	BP	regulation of establishment or maintenance of cell polarity
GO:0032970	BP	regulation of actin filament-based process
GO:0005198	MF	structural molecule activity
GO:0042995	CC	cell projection
GO:0005576	CC	extracellular region
GO:0031012	CC	extracellular matrix
GO:0032153	CC	cell division site
GO:0005622	CC	intracellular
GO:0031975	CC	envelope
GO:0071944	CC	cell periphery

Developmental process and reproduction (423 terms, 931 genes)

GOID	Ontology	Term
GO:0032502	BP	developmental process
GO:0050793	BP	regulation of developmental process
GO:0000003	BP	reproduction
GO:0022414	BP	reproductive process
GO:2000241	BP	regulation of reproductive process

Environmental information processing, response to stimulus and signaling (436 terms, 1161 genes)

GOID	Ontology	Term
GO:0050896	BP	response to stimulus
GO:0023052	BP	signaling
GO:0048583	BP	regulation of response to stimulus
GO:0023051	BP	regulation of signaling
GO:0001816	BP	cytokine production
GO:0001817	BP	regulation of cytokine production
GO:0060300	BP	regulation of cytokine activity
GO:0005125	MF	cytokine activity
GO:0060089	MF	molecular transducer activity
GO:0030545	MF	receptor regulator activity
GO:0043235	CC	receptor complex

Genetic Information process (464 terms, 1944 genes)

GOID	Ontology	Term
GO:0010467	BP	gene expression
GO:0090304	BP	nucleic acid metabolic process
GO:0019219	BP	regulation of nucleobase-containing compound metabolic process
GO:0010468	BP	regulation of gene expression
GO:0005667	CC	transcription factor complex
GO:0008134	MF	transcription factor binding
GO:0030880	CC	RNA polymerase complex
GO:0051983	BP	regulation of chromosome segregation
GO:0036369	BP	transcription factor catabolic process
GO:0001071	MF	nucleic acid binding transcription factor activity
GO:0000988	MF	protein binding transcription factor activity
GO:0051101	BP	regulation of DNA binding
GO:1902415	BP	regulation of mRNA binding
GO:0003676	MF	nucleic acid binding
GO:0060968	BP	regulation of gene silencing
GO:0051276	BP	chromosome organization
GO:0007059	BP	chromosome segregation
GO:0033044	BP	regulation of chromosome organization
GO:0045182	MF	translation regulator activity
GO:0004518	MF	nuclease activity
GO:0003678	MF	DNA helicase activity
GO:0003724	MF	RNA helicase activity

GO:0034061	MF	DNA polymerase activity
GO:0034062	MF	RNA polymerase activity
GO:0008173	MF	RNA methyltransferase activity
GO:0009008	MF	DNA-methyltransferase activity
GO:0019013	CC	viral nucleocapsid
GO:0005694	CC	chromosome
GO:0005634	CC	nucleus
GO:0042575	CC	DNA polymerase complex
GO:0030880	CC	RNA polymerase complex
GO:0032993	CC	protein-DNA complex
GO:0017053	CC	transcriptional repressor complex
GO:0030529	CC	ribonucleoprotein complex

Immune system (171 terms, 455 genes)

GOID	Ontology	Term
GO:0002376	BP	immune system process
GO:0002682	BP	regulation of immune system process

Localization and transport (291 terms, 844 genes)

GOID	Ontology	Term
GO:0051179	BP	localization
GO:0005215	MF	transporter activity
GO:0032879	BP	regulation of localization
GO:1990351	CC	transporter complex
GO:0016247	MF	channel regulator activity
GO:0012505	CC	endomembrane system

Membrane (98 terms, 1223 genes)

GOID	Ontology	Term
GO:0016020	CC	membrane
GO:0061024	BP	membrane organization
GO:0042391	BP	regulation of membrane potential
GO:1903729	BP	regulation of plasma membrane organization
GO:0010549	BP	regulation of membrane disassembly
GO:0090559	BP	regulation of membrane permeability
GO:0003254	BP	regulation of membrane depolarization
GO:0022406	BP	membrane docking
GO:0005604	CC	basement membrane
GO:0032154	CC	cleavage furrow
GO:0031975	CC	envelope

Metabolism (356 terms, 1474 genes)

GOID	Ontology	Term
GO:0008152	BP	metabolic process
GO:0019222	BP	regulation of metabolic process

Organelle (60 terms, (719 genes)

GOID	Ontology	Term
GO:0043226	CC	organelle
GO:0033043	BP	regulation of organelle organization
GO:0006996	BP	organelle organization

System process (68 terms, 298 genes)

GOID	Ontology	Term
GO:0003008	BP	system process
GO:0044057	BP	regulation of system process
GO:0048771	BP	tissue remodeling
GO:0034103	BP	regulation of tissue remodeling
GO:0001503	BP	ossification
GO:0030278	BP	regulation of ossification
GO:0097458	CC	neuron part
GO:0043209	CC	myelin sheath
GO:0043292	CC	contractile fiber

GSIS processes

Associations between the defined GSIS processes and the ancestor terms in GO. BP = Biological Process, MF = Molecular Function, CC= Cellular Components. For each GSIS process, the number of GO terms/pathways and genes associated is reported in brackets.

Calcium-mediated processing (15 terms, 142 genes)

GOID	Ontology	Term
GO:0019722	BP	calcium-mediated signaling
GO:0051592	BP	response to calcium ion
GO:0005509	MF	calcium ion binding
GO:0030899	MF	calcium-dependent ATPase activity
GO:0048306	MF	calcium-dependent protein binding
GO:1901876	BP	regulation of calcium ion binding
GO:0090534	CC	calcium ion-transporting ATPase complex
GO:0048763	MF	calcium-induced calcium release activity
GO:0050848	BP	regulation of calcium-mediated signaling
GO:0097231	BP	cell motility in response to calcium ion
GO:0010857	MF	calcium-dependent protein kinase activity
GO:0051282	BP	regulation of sequestering of calcium ion
GO:0072732	BP	cellular response to calcium ion starvation
GO:1903610	BP	regulation of calcium-dependent ATPase activity
GO:0007223	BP	Wnt signaling pathway, calcium modulating pathway
GO:0030925	BP	calcium incorporation into metallo-oxygen cluster
GO:0046586	BP	regulation of calcium-dependent cell-cell adhesion

GO:0010858	MF	calcium-dependent protein kinase regulator activity
GO:0004198	MF	calcium-dependent cysteine-type endopeptidase activity
GO:0005954	CC	calcium- and calmodulin-dependent protein kinase complex
GO:0007204	BP	positive regulation of cytosolic calcium ion concentration
GO:0051481	BP	negative regulation of cytosolic calcium ion concentration
GO:1901894	BP	regulation of calcium-transporting ATPase activity
GO:0008294	MF	calcium- and calmodulin-responsive adenylate cyclase activity
GO:0004723	MF	calcium-dependent protein serine/threonine phosphatase activity
GO:0008591	BP	regulation of Wnt signaling pathway, calcium modulating pathway
GO:0010859	MF	calcium-dependent cysteine-type endopeptidase inhibitor activity
GO:0075020	BP	calcium or calmodulin-mediated activation of appressorium formation
GO:0008597	MF	calcium-dependent protein serine/threonine phosphatase regulator activity
GO:0048101	MF	calcium- and calmodulin-regulated 3',5'-cyclic-GMP phosphodiesterase activity
GO:0016339	BP	calcium-dependent cell-cell adhesion via plasma membrane cell adhesion molecules
GO:0075103	BP	modulation by host of symbiont calcium or calmodulin-mediated signal transduction
GO:0075133	BP	modulation by symbiont of host calcium or calmodulin-mediated signal transduction
GO:0016338	BP	calcium-independent cell-cell adhesion via plasma membrane cell-adhesion molecules
GO:0075177	BP	regulation of calcium or calmodulin-mediated signal transduction in response to host
GO:0005955	CC	calcineurin complex
GO:0005000	MF	vasopressin receptor activity
GO:0004683	MF	calmodulin-dependent protein kinase activity
GO:0005516	MF	calmodulin binding
GO:0005544	MF	calcium-dependent phospholipid binding

Glycolysis, Glucose processing, Pyruvate metabolism and TCA cycle (29 terms, 179 genes)

GOID	Ontology	Term
GO:0006848	BP	pyruvate transport
GO:0009444	BP	pyruvate oxidation
GO:1902912	CC	pyruvate kinase complex
GO:0004743	MF	pyruvate kinase activity
GO:0047112	MF	pyruvate oxidase activity
GO:0006090	BP	pyruvate metabolic process
GO:0019164	MF	pyruvate synthase activity
GO:0004736	MF	pyruvate carboxylase activity
GO:0045254	CC	pyruvate dehydrogenase complex
GO:0004737	MF	pyruvate decarboxylase activity
GO:0004738	MF	pyruvate dehydrogenase activity
GO:0006849	BP	plasma membrane pyruvate transport
GO:0034604	MF	pyruvate dehydrogenase (NAD+) activity
GO:0050408	MF	[pyruvate kinase]-phosphatase activity
GO:1903302	BP	regulation of pyruvate kinase activity
GO:0050243	MF	pyruvate dehydrogenase (NADP+) activity
GO:0019450	BP	L-cysteine catabolic process to pyruvate
GO:0045250	CC	cytosolic pyruvate dehydrogenase complex
GO:0034603	MF	pyruvate dehydrogenase [NAD(P)+] activity

GO:0052737	MF	pyruvate dehydrogenase (quinone) activity
GO:0019687	BP	pyruvate biosynthetic process from acetate
GO:0043873	MF	pyruvate-flavodoxin oxidoreductase activity
GO:0050244	MF	pyruvate oxidase (CoA-acetylating) activity
GO:0050833	MF	pyruvate transmembrane transporter activity
GO:0009078	BP	pyruvate family amino acid metabolic process
GO:0019272	BP	L-alanine biosynthetic process from pyruvate
GO:0019480	BP	L-alanine oxidation to pyruvate via D-alanine
GO:1902361	BP	mitochondrial pyruvate transmembrane transport
GO:0009079	BP	pyruvate family amino acid biosynthetic process
GO:0019661	BP	glucose catabolic process to lactate via pyruvate
GO:0045253	CC	pyruvate dehydrogenase (lipoamide) phosphatase complex
GO:0004741	MF	[pyruvate dehydrogenase (lipoamide)] phosphatase activity
GO:0010510	BP	regulation of acetyl-CoA biosynthetic process from pyruvate
GO:0004740	MF	pyruvate dehydrogenase (acetyl-transferring) kinase activity
GO:0005477	MF	pyruvate secondary active transmembrane transporter activity
GO:0045249	CC	cytosol pyruvate dehydrogenase (lipoamide) phosphatase complex
GO:0019909	MF	[pyruvate dehydrogenase (lipoamide)] phosphatase regulator activity
GO:0019451	BP	L-cysteine catabolic process to pyruvate, using cysteine dioxygenase
GO:0019910	CC	mitochondrial pyruvate dehydrogenase (lipoamide) phosphatase complex
GO:0019643	BP	reductive tricarboxylic acid cycle
GO:0045239	CC	tricarboxylic acid cycle enzyme complex
GO:0045246	CC	cytosolic tricarboxylic acid cycle enzyme complex
GO:0006097	BP	glyoxylate cycle
GO:0006101	BP	citrate metabolic process
GO:0006107	BP	oxaloacetate metabolic process
GO:0006103	BP	2-oxoglutarate metabolic process
GO:0015140	MF	malate transmembrane transporter activity
GO:0006102	BP	isocitrate metabolic process
GO:0006105	BP	succinate metabolic process
GO:0009361	CC	succinate-CoA ligase complex (ADP-forming)
GO:0015138	MF	fumarate transmembrane transporter activity
GO:0015139	MF	alpha-ketoglutarate transmembrane transporter activity
GO:0006084	BP	acetyl-CoA metabolic process
GO:0016421	MF	CoA carboxylase activity
GO:0005536	MF	glucose binding
GO:0015758	BP	glucose transport
GO:0009749	BP	response to glucose
GO:0042593	BP	glucose homeostasis
GO:0046562	MF	glucose oxidase activity
GO:0061490	BP	glucose import into cell
GO:0006006	BP	glucose metabolic process
GO:0015304	MF	glucose uniporter activity
GO:1990350	CC	glucose transporter complex
GO:0015760	BP	glucose-6-phosphate transport
GO:0004344	MF	glucose dehydrogenase activity

GO:0004346	MF	glucose-6-phosphatase activity
GO:0008877	MF	glucose-1-phosphatase activity
GO:0010827	BP	regulation of glucose transport
GO:2000970	BP	regulation of detection of glucose
GO:0006009	BP	glucose 1-phosphate phosphorylation
GO:0019255	BP	glucose 1-phosphate metabolic process
GO:0051156	BP	glucose 6-phosphate metabolic process
GO:0098708	BP	glucose import across plasma membrane
GO:0004347	MF	glucose-6-phosphate isomerase activity
GO:0046015	BP	regulation of transcription by glucose
GO:0010906	BP	regulation of glucose metabolic process
GO:0042149	BP	cellular response to glucose starvation
GO:0047934	MF	glucose 1-dehydrogenase (NAD+) activity
GO:0047061	MF	glucose-fructose oxidoreductase activity
GO:0047935	MF	glucose 1-dehydrogenase (NADP+) activity
GO:0047938	MF	glucose-6-phosphate 1-epimerase activity
GO:0047936	MF	glucose 1-dehydrogenase [NAD(P)] activity
GO:0004345	MF	glucose-6-phosphate dehydrogenase activity
GO:0005355	MF	glucose transmembrane transporter activity
GO:0047933	MF	glucose-1,6-bisphosphate synthase activity
GO:0008876	MF	quinoprotein glucose dehydrogenase activity
GO:1990576	MF	G-protein coupled glucose receptor activity
GO:2000972	BP	positive regulation of detection of glucose
GO:0032114	BP	regulation of glucose-6-phosphatase activity
GO:0046430	BP	non-phosphorylated glucose metabolic process
GO:0036448	BP	cellular response to glucose-phosphate stress
GO:0047937	MF	glucose-1-phosphate phosphodismutase activity
GO:0005362	MF	low-affinity glucose:sodium symporter activity
GO:0044381	BP	glucose import in response to insulin stimulus
GO:0047468	MF	phosphoglucomutase (glucose-cofactor) activity
GO:0061622	BP	glycolytic process through glucose-1-phosphate
GO:0010170	CC	glucose-1-phosphate adenylyltransferase complex
GO:0008878	MF	glucose-1-phosphate adenylyltransferase activity
GO:0047344	MF	glucose-1-phosphate guanylyltransferase activity
GO:1902659	BP	regulation of glucose mediated signaling pathway
GO:0008524	MF	glucose 6-phosphate:phosphate antiporter activity
GO:0047343	MF	glucose-1-phosphate cytidylyltransferase activity
GO:0008879	MF	glucose-1-phosphate thymidylyltransferase activity
GO:0061612	BP	galactose to glucose-1-phosphate metabolic process
GO:0010474	MF	glucose-1-phosphate guanylyltransferase (GDP) activity
GO:0015152	MF	glucose-6-phosphate transmembrane transporter activity
GO:1902924	BP	poly(hydroxyalkanoate) biosynthetic process from glucose
GO:0052749	MF	glucose-6-phosphate dehydrogenase (coenzyme F420) activity
GO:0089702	MF	undecaprenyl-phosphate glucose phosphotransferase activity
GO:0061513	MF	glucose 6-phosphate:inorganic phosphate antiporter activity
GO:0061547	MF	glycogen synthase activity, transferring glucose-1-phosphate

GO:0061633	BP	transport-coupled glycolytic process through glucose-6-phosphate
GO:0090525	BP	regulation of glycolysis involved in cellular glucose homeostasis
GO:0047399	MF	glucose-1-phospho-D-mannosylglycoprotein phosphodiesterase activity
GO:0036008	BP	sucrose catabolic process to fructose-6-phosphate and glucose-6-phosphate
GO:0036279	BP	positive regulation of protein export from nucleus in response to glucose starvation
GO:0004396	MF	hexokinase activity
GO:0009011	MF	starch synthase activity
GO:0050233	MF	pyranose oxidase activity
GO:0004614	MF	phosphoglucomutase activity
GO:0050190	MF	phosphoglucokinase activity
GO:0050782	MF	galactose uniporter activity
GO:1903299	BP	regulation of hexokinase activity
GO:0004339	MF	glucan 1,4-alpha-glucosidase activity
GO:0004512	MF	inositol-3-phosphate synthase activity
GO:0047684	MF	arylamine glucosyltransferase activity
GO:0033840	MF	NDP-glucose-starch glucosyltransferase activity
GO:0005537	MF	mannose binding
GO:0015761	BP	mannose transport
GO:0015725	BP	gluconate transport
GO:0042946	BP	glucoside transport
GO:0015926	MF	glucosidase activity
GO:0017177	CC	glucosidase II complex
GO:0018321	BP	protein glucuronylation
GO:0005982	BP	starch metabolic process
GO:0009251	BP	glucan catabolic process
GO:0000017	BP	alpha-glucoside transport
GO:0006013	BP	mannose metabolic process
GO:0019253	BP	reductive pentose-phosphate cycle
GO:0035429	BP	gluconate transmembrane transport
GO:0008706	MF	6-phospho-beta-glucosidase activity
GO:0043456	BP	regulation of pentose-phosphate shunt
GO:0080042	MF	ADP-glucose pyrophosphohydrolase activity
GO:0015578	MF	mannose transmembrane transporter activity
GO:0010904	BP	regulation of UDP-glucose catabolic process
GO:0015128	MF	gluconate transmembrane transporter activity
GO:0042947	MF	glucoside transmembrane transporter activity
GO:0005460	MF	UDP-glucose transmembrane transporter activity
GO:0005356	MF	hydrogen:glucose symporter activity
GO:0015573	MF	beta-glucoside transmembrane transporter activity
GO:0090564	MF	protein-phosphocysteine-glucose phosphotransferase system transporter activity
GO:0093001	BP	glycolysis from storage polysaccharide
GO:0006110	BP	regulation of glycolytic process
GO:0061616	BP	glycolytic process from fructose through fructose-6-phosphate
GO:0020015	CC	glycosome
GO:0019682	BP	glyceraldehyde-3-phosphate metabolic process

GTPase and G-protein activity (65 terms, 323 genes)

GOID	Ontology	Term
GO:0005525	MF	GTP binding
GO:0051020	MF	GTPase binding
GO:0003924	MF	GTPase activity
GO:0071521	CC	Cdc42 GTPase complex
GO:0046039	BP	GTP metabolic process
GO:1902773	CC	GTPase activator complex
GO:1990131	CC	Gtr1-Gtr2 GTPase complex
GO:0030695	MF	GTPase regulator activity
GO:0036219	MF	GTP diphosphatase activity
GO:0044549	MF	GTP cyclohydrolase binding
GO:0003933	MF	GTP cyclohydrolase activity
GO:0010292	MF	GTP:GDP antiporter activity
GO:0008728	MF	GTP diphosphokinase activity
GO:0030742	MF	GTP-dependent protein binding
GO:0043087	BP	regulation of GTPase activity
GO:0003935	MF	GTP cyclohydrolase II activity
GO:0043740	MF	GTP cyclohydrolase IIa activity
GO:0070036	MF	GTP-dependent helicase activity
GO:0032794	MF	GTPase activating protein binding
GO:0034211	MF	GTP-dependent protein kinase activity
GO:0015987	BP	GTP synthesis coupled proton transport
GO:0050191	MF	phosphoglycerate kinase (GTP) activity
GO:0060308	MF	GTP cyclohydrolase I regulator activity
GO:0007264	BP	small GTPase mediated signal transduction
GO:0043095	BP	regulation of GTP cyclohydrolase I activity
GO:0051735	MF	GTP-dependent polynucleotide kinase activity
GO:0052754	MF	GTP:coenzyme F420 guanyltransferase activity
GO:0044682	MF	archaeal-specific GTP cyclohydrolase activity
GO:0004613	MF	phosphoenolpyruvate carboxykinase (GTP) activity
GO:0036428	MF	adenosylcobinamide kinase (GTP-specific) activity
GO:0051056	BP	regulation of small GTPase mediated signal transduction
GO:0051730	MF	GTP-dependent polyribonucleotide 5'-hydroxyl-kinase activity
GO:0090113	BP	regulation of ER to Golgi vesicle-mediated transport by GTP hydrolysis
GO:0044082	BP	modulation by symbiont of host small GTPase mediated signal transduction
GO:0060868	BP	regulation of vesicle-mediated transport involved in floral organ abscission by small GTPase mediated signal transduction
GO:0034448	CC	EGO complex
GO:0004383	MF	guanylate cyclase activity
GO:0052621	MF	diguanylate cyclase activity
GO:0047992	MF	hydroxylysine kinase activity
GO:0047341	MF	fucose-1-phosphate guanylyltransferase activity
GO:0047344	MF	glucose-1-phosphate guanylyltransferase activity
GO:0008820	MF	cobinamide phosphate guanylyltransferase activity
GO:0046899	MF	nucleoside triphosphate adenylate kinase activity

GO:0047351	MF	guanosine-triphosphate guanylyltransferase activity
GO:0033263	CC	CORVET complex
GO:0097178	BP	ruffle assembly
GO:0061568	BP	GDP phosphorylation
GO:0034745	CC	APC-IQGAP1-Rac1 complex
GO:0004967	MF	glucagon receptor activity
GO:0034996	CC	RasGAP-Fyn-Lyn-Yes complex
GO:0031291	BP	Ran protein signal transduction
GO:0044600	MF	protein guanylyltransferase activity
GO:0004663	MF	Rab geranylgeranyltransferase activity
GO:0047612	MF	acid-CoA ligase (GDP-forming) activity
GO:0048195	BP	Golgi membrane priming complex assembly
GO:0032045	CC	guanyl-nucleotide exchange factor complex
GO:0005085	MF	guanyl-nucleotide exchange factor activity
GO:0045244	CC	succinate-CoA ligase complex (GDP-forming)
GO:0004776	MF	succinate-CoA ligase (GDP-forming) activity
GO:0043814	MF	phospholactate guanylyltransferase activity
GO:0052618	MF	coenzyme F420-0:L-glutamate ligase activity
GO:0008905	MF	mannose-phosphate guanylyltransferase activity
GO:0072518	MF	Rho-dependent protein serine/threonine kinase activity
GO:0050254	MF	rhodopsin kinase activity
GO:0031681	MF	G-protein beta-subunit binding
GO:0001965	MF	G-protein alpha-subunit binding
GO:0031682	MF	G-protein gamma-subunit binding
GO:0005834	CC	heterotrimeric G-protein complex
GO:0032795	MF	heterotrimeric G-protein binding
GO:0070441	CC	G-protein beta/gamma-Btk complex
GO:0001664	MF	G-protein coupled receptor binding
GO:0070422	CC	G-protein beta/gamma-Raf-1 complex
GO:0097648	CC	G-protein coupled receptor complex
GO:0004930	MF	G-protein coupled receptor activity
GO:0031680	CC	G-protein beta/gamma-subunit complex
GO:0071152	CC	G-protein alpha(q)-synembrin complex
GO:0071153	CC	G-protein alpha(o)-synembrin complex
GO:0071154	CC	G-protein alpha(i)1-synembrin complex
GO:0071155	CC	G-protein alpha(13)-synembrin complex
GO:0031684	BP	heterotrimeric G-protein complex cycle
GO:0031795	MF	G-protein coupled GABA receptor binding
GO:1902712	CC	G-protein coupled GABA receptor complex
GO:1902605	BP	heterotrimeric G-protein complex assembly
GO:0004703	MF	G-protein coupled receptor kinase activity
GO:1990576	MF	G-protein coupled glucose receptor activity
GO:0007186	BP	G-protein coupled receptor signaling pathway
GO:0031683	MF	G-protein beta/gamma-subunit complex binding
GO:0031806	MF	G-protein coupled histamine receptor binding
GO:0038054	MF	G-protein coupled estrogen receptor activity

GO:0001647	MF	G-protein coupled cytokinin receptor activity
GO:0038022	MF	G-protein coupled olfactory receptor activity
GO:0038182	MF	G-protein coupled bile acid receptor activity
GO:0031789	MF	G-protein coupled acetylcholine receptor binding
GO:0038035	BP	G-protein coupled receptor signaling in absence of ligand
GO:0001639	MF	PLC activating G-protein coupled glutamate receptor activity
GO:0015467	MF	G-protein activated inward rectifier potassium channel activity
GO:0008277	BP	regulation of G-protein coupled receptor protein signaling pathway
GO:0044503	BP	modulation of G-protein coupled receptor activity in other organism
GO:0038040	BP	cross-receptor activation within G-protein coupled receptor heterodimer
GO:0038041	BP	cross-receptor inhibition within G-protein coupled receptor heterodimer
GO:0044505	BP	positive regulation of G-protein coupled receptor activity in other organism
GO:0001789	BP	G-protein coupled receptor signaling pathway, coupled to S1P second messenger
GO:1900128	BP	regulation of G-protein activated inward rectifier potassium channel activity
GO:0075087	BP	modulation by host of symbiont G-protein coupled receptor protein signal transduction
GO:0075093	BP	modulation by host of symbiont signal transduction mediated by G-protein beta subunit
GO:0075118	BP	modulation by symbiont of host G-protein coupled receptor protein signal transduction
GO:0075124	BP	modulation by symbiont of host signal transduction mediated by G-protein beta subunit
GO:0075162	BP	regulation of G-protein beta subunit-mediated signal transduction in response to host
GO:0075090	BP	modulation by host of symbiont signal transduction mediated by G-protein alpha subunit
GO:0075096	BP	modulation by host of symbiont signal transduction mediated by G-protein gamma subunit
GO:0075121	BP	modulation by symbiont of host signal transduction mediated by G-protein alpha subunit
GO:0075127	BP	modulation by symbiont of host signal transduction mediated by G-protein gamma subunit
GO:0075156	BP	regulation of G-protein coupled receptor protein signaling pathway in response to host
GO:0075159	BP	regulation of G-protein alpha subunit-mediated signal transduction in response to host
GO:0075165	BP	regulation of G-protein gamma subunit-mediated signal transduction in response to host
GO:0010619	BP	adenylate cyclase-activating glucose-activated G-protein coupled receptor signaling pathway
GO:0044513	BP	envenomation resulting in modulation of G-protein coupled receptor activity in other organism
GO:0086093	BP	G-protein coupled acetylcholine receptor signaling pathway involved in involved in heart process
GO:1903554	BP	G-protein coupled receptor signaling pathway involved in defense response to Gram-negative bacterium
GO:0044514	BP	envenomation resulting in positive regulation of G-protein coupled receptor activity in other organism
GO:0086033	BP	G-protein coupled acetylcholine receptor signaling pathway involved in negative regulation of heart rate

Insulin processing and signaling (30 terms, 185 genes)

GOID	Ontology	Term
GO:0043559	MF	insulin binding
GO:0030073	BP	insulin secretion
GO:0032868	BP	response to insulin
GO:0005158	MF	insulin receptor binding
GO:0005899	CC	insulin receptor complex
GO:1901142	BP	insulin metabolic process
GO:0038020	BP	insulin receptor recycling
GO:0032593	CC	insulin-responsive compartment

GO:0050796	BP	regulation of insulin secretion
GO:0038016	BP	insulin receptor internalization
GO:0005520	MF	insulin-like growth factor binding
GO:0043560	MF	insulin receptor substrate binding
GO:0005009	MF	insulin-activated receptor activity
GO:0030232	CC	insulin control element activator complex
GO:0042568	CC	insulin-like growth factor binary complex
GO:0042567	CC	insulin-like growth factor ternary complex
GO:0005159	MF	insulin-like growth factor receptor binding
GO:0044381	BP	glucose import in response to insulin stimulus
GO:1990418	BP	response to insulin-like growth factor stimulus
GO:0016942	CC	insulin-like growth factor binding protein complex
GO:1900076	BP	regulation of cellular response to insulin stimulus
GO:0048009	BP	insulin-like growth factor receptor signaling pathway
GO:0005010	MF	insulin-like growth factor-activated receptor activity
GO:0005360	MF	insulin-responsive hydrogen:glucose symporter activity
GO:1990314	BP	cellular response to insulin-like growth factor stimulus
GO:0043567	BP	regulation of insulin-like growth factor receptor signaling pathway

Ion transport and homeostasis (84 terms, 290 genes)

GOID	Ontology	Term
GO:0006811	BP	ion transport
GO:0050801	BP	ion homeostasis
GO:0043269	BP	regulation of ion transport
GO:2000021	BP	regulation of ion homeostasis
GO:0015075	MF	ion transmembrane transporter activity

Membrane potential and Ion channels (49 terms, 144 genes)

GOID	Ontology	Term
GO:0034702	CC	ion channel complex
GO:0044325	MF	ion channel binding
GO:0005216	MF	ion channel activity
GO:0022836	MF	gated channel activity
GO:0010359	BP	regulation of anion channel activity
GO:2001257	BP	regulation of cation channel activity
GO:0016247	MF	channel regulator activity
GO:0042391	BP	regulation of membrane potential
GO:0022810	MF	membrane potential driven uniporter activity
GO:0016057	BP	regulation of membrane potential in photoreceptor cell
GO:0016058	BP	maintenance of membrane potential in photoreceptor cell by rhodopsin mediated signaling
GO:0003254	BP	regulation of membrane depolarization
GO:0019227	BP	neuronal action potential propagation
GO:0022814	MF	facilitated diffusion
GO:0001518	CC	voltage-gated sodium channel complex

GO:0005891	CC	voltage-gated calcium channel complex
GO:0008076	CC	voltage-gated potassium channel complex
GO:0022865	MF	transmembrane electron transfer carrier
GO:0046609	MF	voltage-gated sulfate antiporter activity
GO:0008087	CC	light-activated voltage-gated calcium channel complex
GO:0060072	MF	large conductance calcium-activated potassium channel activity
GO:0015291	MF	secondary active transmembrane transporter activity
GO:0086016	BP	AV node cell action potential
GO:0098900	BP	regulation of action potential
GO:0022832	MF	voltage-gated channel activity
GO:0070073	BP	clustering of voltage-gated calcium channels
GO:1990454	CC	L-type voltage-gated calcium channel complex
GO:0070978	BP	voltage-gated calcium channel complex assembly
GO:0015274	MF	organellar voltage-gated chloride channel activity
GO:1990028	MF	intermediate voltage-gated calcium channel activity
GO:1901385	BP	regulation of voltage-gated calcium channel activity
GO:1902941	BP	regulation of voltage-gated chloride channel activity
GO:0008086	MF	light-activated voltage-gated calcium channel activity
GO:1903817	BP	negative regulation of voltage-gated potassium channel activity
GO:1903818	BP	positive regulation of voltage-gated potassium channel activity
GO:0010462	BP	regulation of light-activated voltage-gated calcium channel activity
GO:0044488	BP	modulation of voltage-gated sodium channel activity in other organism
GO:0044360	BP	modulation of voltage-gated potassium channel activity in other organism
GO:0035918	BP	negative regulation of voltage-gated calcium channel activity in other organism
GO:0044492	BP	envenomation resulting in modulation of voltage-gated sodium channel activity in other organism
GO:0044559	BP	envenomation resulting in modulation of voltage-gated potassium channel activity in other organism
GO:0044474	BP	envenomation resulting in negative regulation of voltage-gated calcium channel activity in other organism
GO:1990227	BP	paranodal junction maintenance
GO:0045161	BP	neuronal ion channel clustering
GO:0044736	MF	acid-sensing ion channel activity

Oxidation-reduction and Oxygen-mediated activity (40 terms, 227 genes)

GOID	Ontology	Term
GO:0072593	BP	reactive oxygen species metabolic process
GO:2000377	BP	regulation of reactive oxygen species metabolic process
GO:0015671	BP	oxygen transport
GO:0072592	BP	oxygen metabolic process
GO:0019825	MF	oxygen binding
GO:0032364	BP	oxygen homeostasis
GO:0010242	MF	oxygen evolving activity
GO:0070482	BP	response to oxygen levels
GO:0000302	BP	response to reactive oxygen species
GO:0005344	MF	oxygen transporter activity
GO:2000374	BP	regulation of oxygen metabolic process

GO:1901031	BP	regulation of response to reactive oxygen species
GO:0055114	BP	oxidation-reduction process
GO:1990204	CC	oxidoreductase complex
GO:0016491	MF	oxidoreductase activity
GO:0051341	BP	regulation of oxidoreductase activity
GO:1990042	MF	glycerol dehydrogenase [NAD(P)+] activity
GO:0045272	CC	plasma membrane respiratory chain complex I
GO:0070469	CC	respiratory chain
GO:0008535	BP	respiratory chain complex IV assembly
GO:0034552	BP	respiratory chain complex II assembly
GO:0017062	BP	respiratory chain complex III assembly
GO:0033108	BP	mitochondrial respiratory chain complex assembly
GO:0005753	CC	mitochondrial proton-transporting ATP synthase complex
GO:1903264	MF	nitrate reductase activity involved in anaerobic electron transport chain
GO:0009055	MF	electron carrier activity
GO:0016860	MF	intramolecular oxidoreductase activity
GO:0006119	BP	oxidative phosphorylation

Oxoacid metabolic process and Fatty acid activity (86 terms, 252 genes)

GOID	Ontology	Term
GO:0043436	BP	oxoacid metabolic process
GO:0016833	MF	oxo-acid-lyase activity
GO:0005504	MF	fatty acid binding
GO:0015908	BP	fatty acid transport
GO:0000062	MF	fatty-acyl-CoA binding
GO:0055089	BP	fatty acid homeostasis
GO:0070542	BP	response to fatty acid
GO:0015916	BP	fatty-acyl-CoA transport
GO:0015645	MF	fatty acid ligase activity
GO:0015915	BP	fatty-acyl group transport
GO:0005835	CC	fatty acid synthase complex
GO:0009923	CC	fatty acid elongase complex
GO:0004312	MF	fatty acid synthase activity
GO:0006631	BP	fatty acid metabolic process
GO:0015913	BP	short-chain fatty acid import
GO:1901567	MF	fatty acid derivative binding
GO:0047888	MF	fatty acid peroxidase activity
GO:0005836	CC	fatty-acyl-CoA synthase complex
GO:0015245	MF	fatty acid transporter activity
GO:1901571	BP	fatty acid derivative transport
GO:1903173	BP	fatty alcohol metabolic process
GO:1903174	BP	fatty alcohol catabolic process
GO:0004321	MF	fatty-acyl-CoA synthase activity
GO:0015912	BP	short-chain fatty acid transport
GO:0035337	BP	fatty-acyl-CoA metabolic process

GO:0003997	MF	acyl-CoA oxidase activity
GO:1903175	BP	fatty alcohol biosynthetic process
GO:2000191	BP	regulation of fatty acid transport
GO:0015607	MF	fatty-acyl-CoA transporter activity
GO:0017064	MF	fatty acid amide hydrolase activity
GO:0015246	MF	fatty-acyl group transporter activity
GO:0003995	MF	acyl-CoA dehydrogenase activity
GO:0080132	MF	fatty acid alpha-hydroxylase activity
GO:0042389	MF	omega-3 fatty acid desaturase activity
GO:0045485	MF	omega-6 fatty acid desaturase activity
GO:1990486	BP	anaerobic fatty acid catabolic process
GO:0030733	MF	fatty acid O-methyltransferase activity
GO:0034016	MF	polyenoic fatty acid isomerase activity
GO:0050062	MF	long-chain-fatty-acyl-CoA reductase activity
GO:1901568	BP	fatty acid derivative metabolic process
GO:0030339	MF	fatty-acyl-ethyl-ester synthase activity
GO:0003996	MF	acyl-CoA ligase activity
GO:0052722	MF	fatty acid in-chain hydroxylase activity
GO:1902898	BP	fatty acid methyl ester metabolic process
GO:0019217	BP	regulation of fatty acid metabolic process
GO:0031955	MF	short-chain fatty acid-CoA ligase activity
GO:0015635	MF	short-chain fatty acid transporter activity
GO:0031956	MF	medium-chain fatty acid-CoA ligase activity
GO:1902899	BP	fatty acid methyl ester biosynthetic process
GO:0036125	CC	fatty acid beta-oxidation multienzyme complex
GO:0035338	BP	long-chain fatty-acyl-CoA biosynthetic process
GO:0044535	MF	very-long-chain fatty acyl-CoA oxidase activity
GO:1902321	BP	methyl-branched fatty acid biosynthetic process
GO:0036113	BP	very long-chain fatty-acyl-CoA catabolic process
GO:0015483	MF	long-chain fatty acid transporting porin activity
GO:0008421	MF	long-chain fatty-acyl-glutamate deacylase activity
GO:0015636	MF	short-chain fatty acid uptake transporter activity
GO:0033541	BP	fatty acid beta-oxidation, unsaturated, odd number
GO:0033542	BP	fatty acid beta-oxidation, unsaturated, even number
GO:0080019	MF	fatty-acyl-CoA reductase (alcohol-forming) activity
GO:0047474	MF	long-chain fatty acid luciferin component ligase activity
GO:1902925	BP	poly(hydroxyalkanoate) biosynthetic process from fatty acid
GO:1902322	BP	regulation of methyl-branched fatty acid biosynthetic process
GO:0033544	BP	fatty acid beta-oxidation, unsaturated, even number, epimerase pathway
GO:0033543	BP	fatty acid beta-oxidation, unsaturated, even number, reductase/isomerase pathway
GO:0003987	MF	acetate-CoA ligase activity
GO:0009317	CC	acetyl-CoA carboxylase complex
GO:0008521	MF	acetyl-CoA transporter activity
GO:0035348	BP	acetyl-CoA transmembrane transport
GO:0008775	MF	acetate CoA-transferase activity

Protein tyrosine and serine/threonine kinase activity (104 terms, 297 genes)

GOID	Ontology	Term
GO:0004713	MF	protein tyrosine kinase activity
GO:0030971	MF	receptor tyrosine kinase binding
GO:0030292	MF	protein tyrosine kinase inhibitor activity
GO:0030296	MF	protein tyrosine kinase activator activity
GO:0061097	BP	regulation of protein tyrosine kinase activity
GO:0007169	BP	transmembrane receptor protein tyrosine kinase signaling pathway
GO:0030293	MF	transmembrane receptor protein tyrosine kinase inhibitor activity
GO:0017017	MF	MAP kinase tyrosine/serine/threonine phosphatase activity
GO:1902554	CC	serine/threonine protein kinase complex
GO:0004674	MF	protein serine/threonine kinase activity
GO:0033612	MF	receptor serine/threonine kinase binding
GO:0033867	MF	Fas-activated serine/threonine kinase activity
GO:0004712	MF	protein serine/threonine/tyrosine kinase activity
GO:0030291	MF	protein serine/threonine kinase inhibitor activity
GO:0043539	MF	protein serine/threonine kinase activator activity
GO:0071900	BP	regulation of protein serine/threonine kinase activity
GO:0072518	MF	Rho-dependent protein serine/threonine kinase activity
GO:0016538	MF	cyclin-dependent protein serine/threonine kinase regulator activity
GO:0061575	MF	cyclin-dependent protein serine/threonine kinase activator activity
GO:0007178	BP	transmembrane receptor protein serine/threonine kinase signaling pathway
GO:0090092	BP	regulation of transmembrane receptor protein serine/threonine kinase signaling pathway
GO:0060869	BP	transmembrane receptor protein serine/threonine kinase signaling pathway involved in floral organ abscission
GO:0005963	CC	magnesium-dependent protein serine/threonine phosphatase complex
GO:0008287	CC	protein serine/threonine phosphatase complex
GO:0004722	MF	protein serine/threonine phosphatase activity
GO:0004865	MF	protein serine/threonine phosphatase inhibitor activity
GO:0080163	BP	regulation of protein serine/threonine phosphatase activity
GO:0009400	MF	receptor signaling protein serine/threonine phosphatase activity
GO:0008597	MF	calcium-dependent protein serine/threonine phosphatase regulator activity
GO:0039517	BP	modulation by virus of host protein serine/threonine phosphatase activity

Vesicle-mediated transport and secretion by cell (65 terms, 291 genes)

GOID	Ontology	Term
GO:0032940	BP	secretion by cell
GO:1903530	BP	regulation of secretion by cell
GO:0060471	BP	cortical granule exocytosis
GO:1990504	BP	dense core granule exocytosis
GO:0090522	BP	vesicle tethering involved in exocytosis
GO:0016182	BP	synaptic vesicle budding from endosome
GO:0043317	BP	regulation of cytotoxic T cell degranulation
GO:0055039	CC	trichocyst
GO:0034044	CC	exomer complex

GO:0032195	CC	post-lysosomal vacuole
GO:0097654	CC	platelet SNARE complex
GO:1990502	BP	dense core granule maturation
GO:0055108	BP	Golgi to transport vesicle transport
GO:0044656	BP	regulation of post-lysosomal vacuole size
GO:0070319	CC	Golgi to plasma membrane transport vesicle
GO:0070381	CC	endosome to plasma membrane transport vesicle
GO:0035895	BP	modulation of mast cell degranulation in other organism
GO:0000149	MF	SNARE binding
GO:0031201	CC	SNARE complex
GO:0035495	BP	regulation of SNARE complex disassembly
GO:0005484	MF	SNAP receptor activity
GO:0005802	CC	trans-Golgi network
GO:0030118	CC	clathrin coat
GO:1901950	BP	dense core granule transport
GO:0061110	BP	dense core granule biogenesis
GO:0033363	BP	secretory granule organization
GO:0032253	BP	dense core granule localization
GO:0061109	BP	dense core granule organization
GO:0055107	BP	Golgi to secretory granule transport
GO:1990048	BP	anterograde dense core granule transport
GO:0032255	BP	maintenance of secretory granule location
GO:0033366	BP	protein localization to secretory granule
GO:0032257	BP	maintenance of dense core granule location
GO:2000705	BP	regulation of dense core granule biogenesis
GO:0032254	BP	establishment of secretory granule localization
GO:0032256	BP	establishment of dense core granule localization
GO:0033263	CC	CORVET complex
GO:0070067	CC	syntaxin-6-syntaxin-16-Vti1a complex
GO:0005483	MF	soluble NSF attachment protein activity
GO:0097547	CC	synaptic vesicle protein transport vesicle
GO:1990563	CC	extracellular vesicular exosome complex
GO:0071971	BP	extracellular vesicular exosome assembly
GO:1903551	BP	regulation of extracellular vesicular exosome assembly
GO:1903541	BP	regulation of exosomal secretion
GO:0016192	BP	vesicle-mediated transport
GO:0060627	BP	regulation of vesicle-mediated transport
